# Baohuoside I chemosensitises breast cancer to paclitaxel by suppressing extracellular vesicle/CXCL1 signal released from apoptotic cells

**DOI:** 10.1002/jev2.12493

**Published:** 2024-07-25

**Authors:** Shengqi Wang, Jing Li, Shang Xu, Neng Wang, Bo Pan, Bowen Yang, Yifeng Zheng, Juping Zhang, Fu Peng, Cheng Peng, Zhiyu Wang

**Affiliations:** ^1^ State Key Laboratory of Traditional Chinese Medicine Syndrome, State Key Laboratory of Dampness Syndrome of Chinese Medicine The Second Affiliated Hospital of Guangzhou University of Chinese Medicine Guangzhou China; ^2^ State Key Laboratory of Southwestern Chinese Medicine Resources ChengduUniversity of Traditional Chinese Medicine Chengdu Sichuan China; ^3^ Breast Disease Specialist Hospital of Guangdong Provincial Hospital ofChinese Medicine Guangzhou Guangdong China; ^4^ The Research Center of Integrative Cancer Medicine, Disciplineof Integrated Chinese and Western Medicine The Second Clinical College ofGuangzhou University of Chinese Medicine Guangzhou China; ^5^ Guangdong Provincial Key Laboratory of Clinical Research on Traditional Chinese Medicine Syndrome, Guangdong Provincial Academy of Chinese Medical Sciences Guangdong Provincial Hospital of Chinese Medicine Guangzhou China; ^6^ Guangdong‐Hong Kong‐Macau Joint Lab on Chinese Medicine and Immune Disease Research Guangzhou University of Chinese Medicine Guangzhou China; ^7^ The Research Center for Integrative Medicine, School of Basic Medical Sciences Guangzhou University of Chinese Medicine Guangzhou China; ^8^ Key Laboratory of Drug‐Targeting and Drug Delivery System of the Education Ministry and Sichuan Province West China School of Pharmacy, Sichuan University Chengdu China

**Keywords:** chemotherapy, dying cell signalling, extracellular vesicles, triple‐negative breast cancer, tumour‐associated macrophage

## Abstract

Triple‐negative breast cancer (TNBC) is the most aggressive breast cancer subtype and chemotherapy is the cornerstone treatment for TNBC. Regrettably, emerging findings suggest that chemotherapy facilitates pro‐metastatic changes in the tumour microenvironment. Extracellular vesicles (EVs) have been highly implicated in cancer drug resistance and metastasis. However, the effects of the EVs released from dying cancer cells on TNBC prognosis and corresponding therapeutic strategies have been poorly investigated. This study demonstrated that paclitaxel chemotherapy elicited CXCL1‐enriched EVs from apoptotic TNBC cells (EV‐Apo). EV‐Apo promoted the chemoresistance and invasion of co‐cultured TNBC cells by polarizing M2 macrophages through activating PD‐L1 signalling. However, baohuoside I (BHS) remarkably sensitized the co‐cultured TNBC cells to paclitaxel chemotherapy via modulating EV‐Apo signalling. Mechanistically, BHS remarkably decreased C‐X‐C motif chemokine ligand 1 (CXCL1) cargo within EV‐Apo and therefore attenuated macrophage M2 polarization by suppressing PD‐L1 activation. Additionally, BHS decreased EV‐Apo release by diminishing the biogenesis of intraluminal vesicles (ILVs) within multivesicular bodies (MVBs) of TNBC cells. Furthermore, BHS bound to the LEU104 residue of flotillin 2 (FLOT2) and interrupted its interaction with RAS oncogene family member 31 (RAB31), leading to the blockage of RAB31‐FLOT2 complex‐driven ILV biogenesis. Importantly, BHS remarkably chemosensitised paclitaxel to inhibit TNBC metastasis in vivo by suppressing EV‐Apo^CXCL1^‐induced PD‐L1 activation and M2 polarization of tumour‐associated macrophages (TAMs). This pioneering study sheds light on EV‐Apo^CXCL1^ as a novel therapeutic target to chemosensitise TNBC, and presents BHS as a promising chemotherapy adjuvant to improve TNBC chemosensitivity and prognosis by disturbing EV‐Apo^CXCL1^ biogenesis.

## 1 INTRODUCTION

According to the latest global cancer burden data, breast cancer has been ranked as the most commonly diagnosed cancer and the leading cause of cancer deaths among women globally, with an estimated 2.3 million new cases (24.5%) and 685,000 new deaths (15.5%) in 2020 (Sung et al., 2021). Triple‐negative breast cancer (TNBC), which constitutes 15%−20% of all breast cancers, is the most aggressive breast cancer subtype and has the worst prognosis due to its high recurrence and metastasis rates and the limited therapeutic strategies. The 5‐year survival rate for patients with TNBC is lower than that for other breast cancer subtypes (77% vs. 93%), and patients with metastatic TNBC patients only have a 22% 5‐year survival rate and a 5‐year recurrence rate as high as 80% (Wills et al., 2021). Chemotherapy is the routine treatment for TNBC. According to the 2023 Chinese Society of Clinical Oncology (CSCO) breast cancer guidelines, paclitaxel‐based chemotherapy is the first‐line treatment regimen for TNBC. However, chemotherapy usually shows low pathological complete response (pCR) in TNBC, and acquired chemoresistance severely limits the prognosis of TNBC. For example, only approximately 30% of patients with TNBC achieved pCR to neoadjuvant chemotherapy (Wills et al., 2021). Moreover, emerging evidence suggests that chemotherapy itself is a double‐edged sword. Recent studies have shed new light on the complex interplay between chemotherapy‐induced tumour cell signalling and tumour progression. Although chemotherapy is effective in inhibiting primary tumours, it may accelerate breast cancer metastasis through poorly defined mechanisms (Karagiannis et al., 2017; Li et al., 2022; Wills et al., 2021). Neoadjuvant chemotherapy with paclitaxel can reportedly induce pro‐metastatic changes in the tumour microenvironment (TME) of breast cancer patients (Karagiannis et al., 2017). Thus, there is an urgent need to investigate the molecular mechanisms underlying chemotherapy‐induced breast cancer progression and to develop more effective therapeutics to improve the TNBC prognosis.

Billions of cells die in the human body every day (Luo et al., 2021). Cell death modulation and effective clearance are fundamental processes for immune homeostasis maintenance and neoplastic disease prevention (Boada‐Romero et al., 2020). Notably, cell death‐associated studies over the past decades have primarily focused on death modes and underlying molecular mechanisms, especially the various modes of programmed cell death. To date, over 10 cell death modes (such as apoptosis, necroptosis, autophagy, pyroptosis, ferroptosis and cuproptosis) and multiple death receptors/pathways have been identified (Tang et al., 2019). These achievements have revolutionized the development of anti‐cancer drugs that directly activate the cell death machinery (Tong et al., 2022). It should be noted that dying cells, which are not inactive entities awaiting removal, can release intracellular components as dead signals that actively influence the fate of surrounding cells or tissues (Medina et al., 2020). Dead signals released by dying cells encompass various molecules including metabolites, proteins, nucleic acids, ion signals, cytokines, chemokines and extracellular vesicles (EVs). These dead signals are closely implicated in modulating cancer biological behaviours; they not only function as survival signals to favour cancer growth (Jiang et al., 2020) but also act as crucial modulators regulating tumour immunogenicity, inflammatory infiltration, and anti‐cancer immune responses in the TME. For example, dying colon tumour cells released increased ATP that triggered P2 × 4 to mediate the mTOR‐dependent pro‐survival and anti‐apoptotic programs in neighbouring cancer cells (Schmitt et al., 2022). Necroptosis‐induced CXCL1 and Mincle signalling were found to promote macrophage‐induced adaptive immune suppression and thereby enabled the oncogenesis and progression of pancreatic ductal adenocarcinoma (Seifert et al., 2016). Chemotherapy inevitably leads to the death of numerous cancer cells within a short time and thus, to the release of a massive number of dying signals. An increasing number of studies have also indicated that multiple chemotherapeutics may inhibit primary tumour growth but facilitate cancer immune escape and metastasis (Bellomo et al., 2022; Karagiannis et al., 2017; Keklikoglou et al., 2019; Wills et al., 2021). For example, gemcitabine initially slowed down pancreatic cancer progression but eventually promoted metastasis by triggering neutrophil infiltration (Bellomo et al., 2022). Doxorubicin was also shown to inhibit the primary tumour growth of TNBC while accelerating its pulmonary metastasis by priming the lung pre‐metastatic niche (PMN) (Wills et al., 2021). However, the precise mechanisms of chemotherapy‐elicited dead signals in modulating tumour metastasis and chemoresistance have been poorly revealed and deserve further exploration.

Exosomes are a subset of EVs that have an average diameter of approximately 100 nm. The main biological function of exosomes is to mediate intercellular communication by transferring various constituents (e.g., metabolites, nucleic acids, proteins, lipids, and amino acids). Exosome biogenesis begins with the endocytosis process, where cellular constituents are engulfed and enclosed within early endosomes. Subsequently, these early endosomes mature into multivesicular bodies (MVBs), encapsulate specific cellular cargo to form intraluminal vesicles (ILVs), and eventually release the ILVs as exosomes into the extracellular environment when MVBs fuse with the plasma membrane (Kalluri & LeBleu, 2020). Notably, EVs have emerged as central regulators of cancer progression and metastasis by transferring the cargo molecules to recipient cells, altering their phenotype and function (Villamizar et al., 2021). The biological effects of cancer cell‐derived EVs have been widely investigated in recent studies. For example, in ovarian cancer, exosomal miR‐141 from cancer cells transformed stromal fibroblasts into pro‐inflammatory cancer‐associated fibroblasts, promoting the formation of a PMN (Mo et al., 2023). In breast cancer, exosomal CD274 from cancer cells suppressed T cell‐mediated killing activity and enhanced tumour growth (Yang et al., 2018). Additionally, colorectal cancer‐derived exosomal miR‐934 accelerated liver metastasis by promoting macrophage M2 polarization (Zhao et al., 2020). In summary, cancer cell‐derived EVs not only directly transfer the malignant traits of cancer cells to the surrounding normal cells or distant organs, but also suppress anti‐cancer immunity by inducing cytotoxic T‐cell exhaustion and activating immunosuppressive cells (de Vrij et al., 2015; Taylor & Gercel‐Taylor, 2011; Xu et al., 2020). Nevertheless, the vast majority of current EV‐related studies have focused on EVs released from viable cancer cells (Li et al., 2019; Wortzel et al., 2019), whereas the biological function of dying cancer cell‐released EVs remains poorly understood. In recent years, emerging evidence has suggested that chemotherapy can enhance EV secretion from cancer cells to accelerate pulmonary metastasis by priming the PMN (Keklikoglou et al., 2019; Wills et al., 2021). Macrophages are the most abundant immune cells infiltrated in the TME of breast cancer, and have been demonstrated to meditate breast cancer metastasis and chemoresistance. However, it remains unclear whether chemotherapy‐elicited EVs from tumours participated in the metastasis and chemoresistance of breast cancer by modulating tumour associated macrophages (TAMs) in the TME. Additionally, as one of the most abundant chemokines in the TME, CXCL1 was found to promote the immune escape and chemoresistance of breast cancer through multiple mechanisms, including activating autophagy, promoting the self‐renewal property of cancer stem cells (CSCs), and inducing immunosuppressive cell infiltration and PMN formation (Li et al., 2021). Clinically, CXCL1 elevation was also closely correlated with poor chemosensitivity and recurrence‐free survival of breast cancer (Korbecki et al., 2023; Zou et al., 2014). Interestingly, it has been reported that chemotherapy‐elicited necroptosis CXCL1 signals can accelerate pancreatic oncogenesis and progression by activating PD‐L1 expression on TAMs (Seifert et al., 2016) to establish immunosuppressive TME. Based on the above information, we speculated that chemotherapy may also modulate CXCL1 cargo in EVs and therefore activate TAM/PD‐L1 signalling to promote breast cancer chemoresistance and metastasis.

Baohuoside I (BHS) is a natural prenylflavonoid and the main bioactive component of *Epimedium brevicornum Maxim*, a traditional Chinese herb that, for thousands of years, has been used for the treatment of fractures and bone and joint diseases with excellent therapeutic effects. BHS possesses multiple biological and pharmacological properties, including anti‐osteoporotic, anti‐inflammatory, antioxidant, invigorative, and neuroprotective effects. Notably, accumulating evidence also suggests that BHS is a promising anti‐cancer drug for multiple malignancies including breast cancer (Sun et al., 2020; Wang et al., 2020). The anti‐cancer mechanisms of BHS involve inducing cell cycle arrest and apoptosis, inhibiting neoangiogenesis, suppressing CSCs, modulating miRNAs, suppressing autophagy, and modulating the phenotype and function of various immune cells (Chen et al., 2016). Our previous study showed that BHS inhibits breast cancer growth and metastasis by suppressing TAM/CXCL1‐induced self‐renewal of CSCs (Wang et al., 2020). In addition to these direct cancer inhibition effects, recent studies revealed that BHS has the potential to act as an adjuvant agent to chemosensitise malignancies with other cytotoxic drugs (Wu et al., 2012; Zhou et al., 2023). For example, BHS synergistically interacted with cisplatin to chemosensitise ovarian cancer by suppressing autophagy via downregulating the HIF‐1α/ATG5 axis (Zhou et al., 2023). BHS also potentiated paclitaxel‐induced apoptosis in human melanoma by inhibiting TLR4 signalling (Wu et al., 2012). Despite these reports, it remains unknown whether BHS can exert anti‐cancer and chemosensitising effects by modulating EVs, and this merits further investigation.

In this study, we systematically demonstrated that BHS suppressed EV‐Apo secretion from paclitaxel‐treated TNBC cells, and therefore synergistically suppressed the growth and metastasis of TNBC by inhibiting CXCL1/TAM/PD‐L1 signalling activated by EV‐Apo. Our study not only uncovers CXCL1 as a key signalling molecule in EV‐Apo mediating chemotherapy‐induced resistance and metastasis, but also highlights BHS as a promising EV‐Apo clearance agent to improve TNBC chemosensitivity and prognosis.

## 2 MATERIALS AND METHODS

### 2.1 Cell lines and culture

TNBC 4T1 cells and Raw264.7 macrophages were purchased from KeyGen Biotech (Nanjing, China). 4T1‐Luc cells were obtained by transfecting 4T1 cells with the lentiviral luciferase reporter plasmid. The human acute monocytic leukaemia cell line THP1 and the human TNBC cell line MDA‐MB‐231 were obtained from the American Type Culture Collection. To induce attachment and differentiation of THP1 monocytes into macrophages, 100 ng/mL phorbol‐12‐myristate‐13‐acetate (PMA) (Sigma–Aldrich, Missouri, USA) was employed. FBS was replaced with exosome‐depleted FBS when the culture medium was used for exosome isolation. For the co‐culture of breast cancer cells and macrophages, the transwell co‐culture system was used as previously described (Wang et al., 2020).

### 2.2 EV isolation, observation, and concentration and size detection

EVs were isolated from the culture medium of breast cancer cells using a differential ultracentrifugation method (Song et al., 2019) or utilizing a low‐speed centrifugation method with RiboTM Exosome Isolation Reagent (C10130‐2, Ribo Biotech, Guangzhou, China) according to the kit instructions. For the differential ultracentrifugation method, the medium was centrifuged at 300 ×*g* for 10 min to remove cells. Then, the supernatant was centrifuged at 2000 × g for 10 min, 10,000 × *g* for 30 min, filtered through a 0.22 µm filter, and pelleted by ultracentrifugation at 100,000 × *g* for 70 min using the Beckman L‐100K ultracentrifuge (Beckman Coulter, Brea, CA, USA). The EV pellet was washed in cold PBS and collected by ultracentrifugation again at 100,000 × *g* for 70 min. Finally, the EV pellet was resuspended in PBS or lysis buffer before further analysis. For EV‐Apo and isolation, 4T1 or MDA‐MB‐231 cells were treated with 1 µM paclitaxel (MB1178, Meilun Biotechnology, Dalian, China) for 24 h. Then, the cells were harvested and stained with Annexin V‐FITC solution (70‐APCC101‐100, MultiSciences, Hangzhou, China) for 5 min. The Annexin V‐FITC‐positive cells were selected by flow cytometry sorting using a FACS Aria III flow cytometer (BD Biosciences, Franklin Lakes, NJ, USA) and cultured in exosome‐depleted medium for 24 h. Then, the culture medium was harvested to isolate EV‐Apo. Similarly, EV‐Apo^BHS^ was isolated from the culture medium of apoptotic 4T1 or MDA‐MB‐231 cells induced by BHS (>99% purity, MB6526, Meilun Biotechnology) and paclitaxel combination treatment. EV‐alive were directly isolated from the culture medium of the untreated 4T1 cells. A transmission electron microscope (TEM) was used to observe the lipid bilayer structures of EVs (Song et al., 2019). A Flow NanoAnalyzer (NanoFCM Inc., Xiamen, China) was used to measure the size distribution and concentration of EVs (Cespedes et al., 2022).

### 2.3 Western blotting

The protein lysates were applied to SDS‐PAGE, transferred to a PVDF membrane (Millipore, Billerica, MA, USA), and probed with primary antibodies for Alix (12422‐1‐AP, Proteintech, Wuhan, China), TSG101 (67381‐1‐Ig, Proteintech), CD81 (66866‐1‐Ig, Proteintech), calnexin (ab133615, Abcam, Cambridge, MA, USA), Annexin V (11060‐1‐AP, Proteintech), E‐cadherin (20874‐1‐AP, Proteintech), N‐cadherin (22018‐1‐AP, Proteintech), vimentin (10366‐1‐AP, Proteintech), GAPDH (5174, CST, Boston, MA, USA), PD‐L1 (DF6526, Affinity, Changzhou, China), FLOT2 (66881‐1‐Ig, Proteintech), and β‐actin (4970S, CST).

### 2.4 Cell proliferation and colony formation assays

For the cell proliferation assay, cells were treated as indicated and cell viability was analysed using the CCK8 Kit (C0038, Beyotime Biotechnology, Hangzhou, China). For the colony formation assay, cells were seeded in a 6‐well plate at a density of 1000 cells per well. After attachment, cells were treated as indicated and then continued to be cultured for 1 week. The resultant colonies were fixed with 4% paraformaldehyde and then stained with Coomassie Blue.

### 2.5 Wound healing and transwell assays

The wound healing and transwell assays were conducted as previously described (Huang et al., 2020) to measure the migration and invasion ability changes of breast cancer cells after the indicated exogenous interventions, respectively.

### 2.6 Cell apoptosis detection

Cell apoptosis of breast cancer cells after the indicated treatment was measured by the NovoCyte flow cytometer (ACEA Biosciences, CA, USA) using the Annexin V‐FITC/PI Apoptosis Detection Kit (KGA107, KeyGEN BioTECH) according to the manufacturer's protocol.

### 2.7 Flow cytometry

The M2 phenotype polarization of macrophages was identified by analysing surface markers using flow cytometry. Briefly, macrophages were treated as indicated, harvested, washed and resuspended in PBS solution. For the phenotypic analysis of Raw264.7 macrophages, cells were incubated with an F4/80‐APC antibody (17‐4801‐82, eBioscience, Waltham, MA, USA) and a CD206‐PE antibody (141705, Biolegend, San Diego, CA, USA) for 30 min at 37°C. For the phenotypic analysis of THP1 macrophages, cells were treated as indicated, harvested and incubated with an F4/80‐APC antibody (17‐4801‐82, Invitrogen) and a CD163‐PE antibody (12‐1639‐42, eBioscience) for 30 min at 37°C. Next, the cells were washed once with PBS, resuspended and subjected to flow cytometry analyses using the NovoCyte flow cytometer (ACEA Biosciences). For the phenotype analyses of primary macrophages isolated from mouse 4T1‐Luc xenografts, cells were incubated with CD45‐PE‐Cy7 (25‐0451‐82, eBioscience), F4/80‐APC (17‐4801‐82, eBioscience), and CD206‐PE (141705, Biolegend) antibodies. For PD‐L1 expression analyses of primary macrophages, cells were incubated with CD45‐PE‐Cy7 (25‐0451‐82, eBioscience), F4/80‐FITC (SC‐71085, Santa Cruz), and PD‐L1‐APC (124312, Biolegend) antibodies. For PD‐L1 expression analyses of primary macrophages, cells were incubated with CD45‐PE‐Cy7 (25‐0451‐82, eBioscience), F4/80‐FITC (SC‐71085, Santa Cruz), and PD‐L1‐APC (124312, Biolegend) antibodies. For granzyme B and perforin production activity detection, cells were incubated with the APC/Cyanine7‐conjugated CD8 antibody (100714, Biolegend), PE‐conjugated perforin antibody (12‐9392‐82, eBioscience) and APC‐conjugated granzyme B antibody (17‐8898‐82, eBioscience) antibodies. Subsequently, cells were washed once with PBS and subjected to a flow cytometry analysis.

### 2.8 Macrophage phagocytosis assay

Briefly, EVs were labelled with the PKH67 green fluorescent cell linker (2 µM, MINI67, Sigma–Aldrich, Missouri, USA). The labelled EVs were collected by ultracentrifugation, washed with PBS and collected again by ultracentrifugation. Then, Raw264.7 macrophages were treated with PKH67‐labeled EVs for 1−4 h. After washing with PBS three times, the Raw264.7 macrophages were harvested and subjected to flow cytometry to measure their green fluorescence intensities. To visualize the phagocytotic process of EVs by macrophages, macrophages were treated with the PKH67‐labeled EVs, washed, fixed, permeabilized, and then incubated with Actin‐Red (5 U/mL, KGMP0012, KeyGEN) for 20 min. Finally, the cells were observed using an LSM710 confocal microscope (Zeiss, Oberkochen, Germany).

### 2.9 ELISA

ELISA assay was conducted to detect CXCL1 concentrations in different EVs. Briefly, equal quantities of EVs were lysed by RIPA and sonication. The CXCL1 concentrations in the lysed EVs were detected using the CXCL1 ELISA Kit (USCN Business, Wuhan, China) according to the kit instructions.

### 2.10 Immunofluorescence assay

An immunofluorescence analysis was conducted as previously described (Huang et al., 2020). The following primary antibodies were used in the immunofluorescence assay: PD‐L1 (DF6526, Affinity), CXCL1 (AF5403, Affinity), CD81 (66866‐1‐Ig, Proteintech), biotin (55648‐1, Signalway antibody, MA, USA), FLOT2 (66881‐1‐Ig, Proteintech), CD63 (143921, Biolegend), CD204 (DF6694, Affinity), TSG101 (67381‐1‐Ig, Proteintech) and CD34 (AF5149, Affinity). The following secondary antibodies were used in the immunofluorescence assay: Alexa Fluor 555 conjugated‐anti‐rabbit IgG (4413S, CST), Alexa Fluor 488 conjugated‐anti‐rabbit IgG (4412S, CST), Alexa Fluor 555 conjugated‐anti‐mouse IgG (A21422, Thermo Fisher, Waltham, MA, USA), FITC conjugated‐anti‐Rat IgG (SC‐2011, Santa Cruz), Alexa Fluor 488 conjugated‐anti‐mouse IgG (4408S, CST), Alexa Fluor 647 conjugated‐anti‐rabbit IgG (4414S, CST), and Dylight 649 conjugated‐anti rat IgG (RS23640, ImmunoWay Biotechnology, Plano, TX, USA). Fluorescence images were obtained using an LSM710 confocal microscope or an N‐SIM/N‐STORM inverted fluorescence microscope (Nikon, Tokyo, Japan).

### 2.11 QPCR

QPCR was conducted as previously described (Huang et al., 2020). The primer sequences were as follows: 5′‐GCTCCAAAGGACTTGTACGTG‐3′ (forward) and 5′‐TGATCTGAAGGGCAGCATTTC‐3′ (reverse) for mouse *PD‐L1* and 5′‐GGAGGGGGTTGAGGTGTT‐3′ (forward) and 5′‐GTGTGCACTTTTATTGGTCTCAA‐3′ (reverse) for mouse *β‐actin*. The 2^−ΔΔCt^ method was used to calculate the relative gene expression levels among groups.

### 2.12 Double luciferase reporter gene assay

To investigate the *PD‐L1* promoter activity changes of macrophages after the indicated treatment, a double luciferase reporter gene assay was conducted as previously described (Huang et al., 2020) using the Secrete‐Pair Dual Luminescence Assay Kit (LF031, Genecopeia, MD, USA). The *PD‐L1* promoter plasmid (MPRM25392‐PG04, Genecopeia) was transfected into Raw264.7 cells.

### 2.13 Plasmid transfection

All plasmids used in this study were purchased from Dahong Biosciences (Guangzhou, China) and transfected into cells using the Vigenefection regnant (FH880806, Vigene Biosciences). The CXCL1 recombinant plasmid with a C‐terminal FLAG tag (CH882537) was used to generate 4T1/rCXCL1‐Flag cells. shRNA plasmids for Alix (SHNM_001164677), Flag‐tagged FLOT2 recombinant plasmids (OENM_001040403) and Flag‐tagged FLOT2^L104A^ mutation plasmids (HY22968) were used to generate 4T1/shAlix cells, 4T1/FlOT2‐OE, and 4T1/FLOT2^L104A^ cells, respectively.

### 2.14 TEM observation of ILVs

ILVs located in MVBs were visualized by TEM. Briefly, cells were treated as indicated, washed with PBS, and fixed with an osmic acid solution for 2 h. Then, the cells were dehydrated, embedded and sectioned. The sections were stained with 2% acetate double oxygenic uranium and 3% citrate lead, and subjected to TEM observation using a JEM‐1200EX electron microscope (JEOL, Tokyo, Japan).

### 2.15 BHS‐pull‐down‐MS assay

BHS‐Biotin was synthesized by Xian Ruixi Biotechnology Co., Ltd. The structure of BHS‐Biotin was characterized by nuclear magnetic resonance hydrogen spectrum (1H NMR) and mass spectroscopy. A BHS‐pull‐down‐MS assay was conducted by Guangzhou Tianjun Biotechnology Co., Ltd. to identify the binding proteins of BHS. Briefly, streptavidin magnetic beads (21344, Thermo‐Fisher Scientific, Waltham, MA) were incubated with biotin or BHS‐Biotin for 1 h, and washed with RIPA lysis buffer three times. Then, the cell lysates of untreated or 5 µM BHS‐Biotin‐treated breast cancer 4T1 cells were prepared and incubated with biotin‐coupled magnetic beads or BHS‐Biotin‐coupled magnetic beads for 2 h. Next, the supernatants were discarded, and the remaining magnetic beads were washed with RIPA lysis buffer five times. Then, the proteins bound to the magnetic beads were eluted and trypsinized. The peptides were identified using tandem mass spectrometry (MS) with an Orbitrap mass spectrometer (Q Exactive, ThermoFisher). The obtained MS raw files were submitted to the MaxQuant software for protein identification and quantification. Proteins with a fold change larger than 2.0 or unique to the BHS‐Biotin treatment group were selected as differentially expressed proteins (DEPs).

### 2.16 Cellular thermal shift assay (CETSA)

CETSA assay was performed as previously described (Zheng et al., 2023) to investigate the thermal stability change of FLOT2 in 4T1 cell lysate after BHS treatment. Briefly, 4T1 cells were treated with 5 µM BHS for 3 h. Then, the BHS‐treated and untreated 4T1 cells were harvested, freeze‐thawed and lysed. The lysates of BHS‐treated cells were exposed to 5 µM BHS for 30 min at room temperature. Then, the cell lysates were heated at different temperatures ranging from 55 to 85°C for 3 min, cooled to room temperature, and centrifuged to separate the supernatants for immunoblot analysis.

### 2.17 Computer‐aided virtual docking between BHS and FLOT2

In silico virtual docking was conducted using the Biovia Discovery Studio 2016 software (BIOVIA, San Diego, CA, USA). The 3D structure of FLOT2 was acquired from an AlphaFold structure prediction (Jumper et al., 2021; Varadi et al., 2022) and the binding site was set on the SPFH domain of FLOT2 since the ^104^LQTL^107^ motif in the SPFH domain of FLOT2 is responsible for FLOT2 interactions (Wei et al., 2021). Flexible docking of BHS to FLOT2 and the subsequent virtual alanine scan protocols were performed as previously described (Zheng et al., 2023). Briefly, the BHS molecule structure was prepared and minimized before the flexible docking protocol was performed. After BHS was successfully docked into FLOT2, a virtual alanine scan procedure was performed to analyse potential amino acids accounting for BHS‐FLOT2 binding.

### 2.18 Co‐immunoprecipitation (Co‐IP) assay

Co‐IP assay was conducted using the Pierce Co‐Immunoprecipitation Kit (no. 26149, Thermo Fisher, Hudson, NH, USA) according to the manufacturer's instructions. Primary antibodies against biotin (55648‐1, Signalway antibody), RAB31 (16182‐1‐AP, Proteintech), Flag (M185‐3L, MBL International Corporation, Woburn, MA, USA) and FLOT2 (66881‐1‐Ig, Proteintech) were used in this assay.

### 2.19 Animal experiments

The animal study was approved by the Institutional Animal Care and Use Committee of Guangdong Provincial Hospital of Chinese Medicine (No. 2021073). For 4T1‐Luc xenograft model establishment, 2 × 10^6^ 4T1‐Luc cells were inoculated subcutaneously into the mammary fat pads of 6‐week‐old female NSG mice or Balb/c mice. The NSG mice were randomly divided into seven groups, namely, saline group, paclitaxel group (10 mg/kg/3d), BHS^high^ group (20 mg/kg/d), paclitaxel (PTX)+BHS^low^ group (10 mg/kg/d), PTX+BHS^high^ group (20 mg/kg/d), PTX+GW4869 group (1.25 mg/kg/d), and PTX+BHS^high^+EV‐Apo^rCXCL1‐Flag^ group (EV‐Apo^rCXCL1^). The Balb/c mice were randomly divided into seven groups, namely, saline group, PTX group (10 mg/kg/3d), BHS group (20 mg/kg/d), PTX+BHS group, PTX+BHS+EV‐Apo group, PTX+BHS+EV‐Apo^shCXCL1^ group, and PTX+BHS+EV‐Apo^rCXCL1^ group. PTX, BHS and GW4869 were administrated intraperitoneally while EVs were administrated by a peritumoral injection with a dose of 200 µg/kg/3d. The mice were imaged using an IVIS Lumina XR in vivo imaging system (PerkinElmer, MA, USA) to monitor tumour growth and metastasis. At the end of the animal experiments, the mice were euthanized, and the primary cells were isolated from tumours or lung metastatic foci by mechanical methods, and subjected to macrophage phenotypic analysis or autophagic activity detection. HE staining was applied as previously reported (Wang et al., 2020) to investigate the lung metastasis differences among different groups. For the sorting of TAMs, primary cells were isolated from fresh mammary tumours by mechanical methods and stained with the F4/80‐APC antibody (17‐4801‐82, eBioscience). Then, the primary macrophages were subjected to FACS sorting. A TUNEL cell apoptosis detection kit (Green fluorescence, C1088, Beyotime, Shanghai, China) was used to label apoptotic tumour cells in vivo. Mouse peripheral blood mononuclear cells (PBMCs) were isolated using the lymphocyte separation medium (TBD Sciences, Tianjin, China) via density gradient centrifugation according to the kit instructions. Subsequently, the cytotoxic effect of BHS on PBMCs was examined using the CCK8 assay.

### 2.20 Circulating tumour cell (CTC) detection

The number of CTCs in the blood of tumour‐bearing mice was measured by detecting the relative expression levels of the luciferase gene derived from breast cancer 4T1‐Luc cells. Genomic DNA was extracted from mouse peripheral blood and measured by QPCR using the following primers: 5′‐GCTCAGCAAGGAGGTAGGTG‐3′ (forward) and 5′‐TCTTACCGGTGTCCAAGTCC‐3′ (reverse) for luciferase.

### 2.21 Autophagic activity detection

Primary tumour cells were isolated from both the mammary tumour tissues and lung metastatic foci. The isolated cells were transfected with mRFP‐GFP‐LC3 adenoviral vectors (HanBio Technology, Shanghai, China) and seeded onto cell climbing slices in 24‐well plates. Following cell adhesion, the autophagic flux was visualized using an LMS710 confocal microscope. Autolysosomes were identified by their free red fluorescence (mRFP), whereas autophagosomes were distinguished by yellow fluorescence due to the colocalization of mRFP and GFP.

### 2.22 Statistical analysis

Data are presented as the mean ± SD. Student's *t*‐test and one‐way ANOVA were used for comparisons among groups. Levene's test of equality of variances was used to assess the assumption of homogeneity of variance. Data with repeated measurements were analysed by repeated‐measures ANOVA followed by post‐hoc test. *n* value represents the number of biological repeats or animals. *p *< 0.05 was considered statistically significant.

## 3 RESULTS

### 3.1 EV‐Apo promotes the invasion and chemoresistance of breast cancer cells co‐cultured with macrophages

**FIGURE 1:**
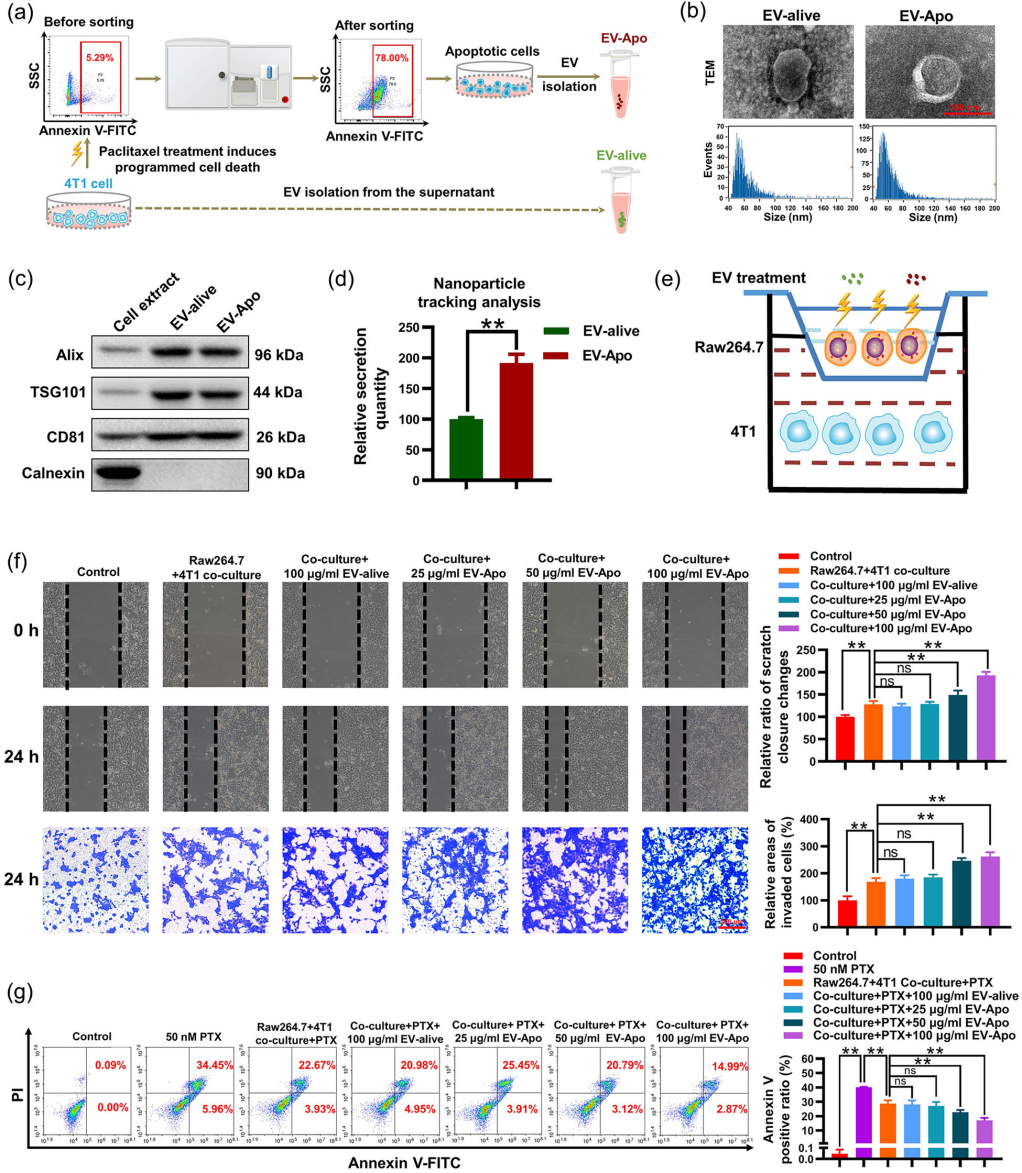
EV‐Apo promotes the invasion and chemoresistance of breast cancer cells co‐cultured with macrophages. (a) A schematic diagram of EV‐Apo and EV‐alive isolation procedures. (b) Representative TEM images of EV‐Apo and EV‐alive as well as their size distribution analysis by a Flow NanoAnalyzer. (c) Expression of exosome markers detected by Western blotting. *n* = 3. (d) Comparison of the secretion quantity difference between EV‐Apo and EV‐alive through nanoparticle tracking analysis (NTA). *n* = 3. (e) A schematic diagram of the co‐culture system. 4T1 cells and Raw264.7 cells were co‐cultured using the transwell co‐culture system. (f) Cell migration and invasion assays of 4T1 cells cultured alone or co‐cultured with Raw264.7 cells. The cells were treated as indicated for 24 h. *n* = 3. (g) Apoptosis rates of 4T1 cells detected by Annexin V‐FITC/PI staining and flow cytometry. 4T1 cells were cultured alone or co‐cultured with Raw264.7 cells and treated as indicated for 48 h. *n* = 3. ^**^ *p* < 0.01.

A paclitaxel‐based regimen is the first‐line strategy for TNBC chemotherapy (Lohard et al., 2020). Therefore, paclitaxel was selected to induce apoptosis in the mouse 4T1 TNBC cell line. As shown in Figure 1(a), the Annexin V‐FITC^+^ apoptotic population of 4T1 cells was first enriched by flow cytometry and continued to be cultured in the exosome‐depleted medium for 24 h. EV‐Apo was further isolated from the supernatants of apoptotic 4T1 cells. EV‐alive was isolated from the supernatants of untreated 4T1 cells. For EV characterization, TEM images suggested that both EV‐Apo and EV‐alive displayed a lipid bilayer structure. The nanoparticle analysis showed that the average diameter is 63.58 nm for EV‐Apo and 64.43 nm for EV‐alive (Figure 1b). Additionally, both EVs exhibited elevated expression of exosome‐positive markers including Alix, TSG101, and CD81, while exhibiting little expression of the exosome‐negative marker calnexin (Figure 1c). The aforementioned results suggest the successful isolation of both EV‐alive and EV‐Apo. Additionally, both NanoFCM (Figure 1b) and BCA analysis (Figure 1d) showed that paclitaxel treatment significantly elevated the quantity of EVs secreted from 4T1 cells. Next, the direct effects of EV‐Apo and EV‐alive on breast cancer cells were investigated. As shown in Figure S1, both EV‐Apo and EV‐alive (100 µg/mL) exhibited minimal effects on breast cancer cell proliferation, migration and invasion. As is well known, macrophages are the most abundant immune cell type infiltrated in the TME of breast cancer (Wang et al., 2021). Additionally, macrophages possess powerful phagocytotic activity, which represents an efficient way for EV uptake. Therefore, the effects of EV‐Apo and EV‐alive on breast cancer cells were further investigated in the co‐culture system with macrophages (Figure 1e). Interestingly, EV‐Apo (50−100 µg/mL) treatment significantly promoted the migration and invasion of breast cancer 4T1 cells in the presence of Raw264.7 macrophages, whereas EV‐alive exhibited minimal effects on that (Figure 1f). Additionally, EV‐Apo (50−100 µg/mL) treatment increased apoptosis resistance of co‐cultured 4T1 cells to paclitaxel, whereas EV‐alive did not (Figure 1g). Altogether, these results suggest that EV‐Apo accelerates breast cancer cell invasion and paclitaxel chemoresistance in the presence of macrophages.

### 3.2 BHS chemosensitises breast cancer cells via modulating paclitaxel‐induced EV‐Apo signalling

**FIGURE 2:**
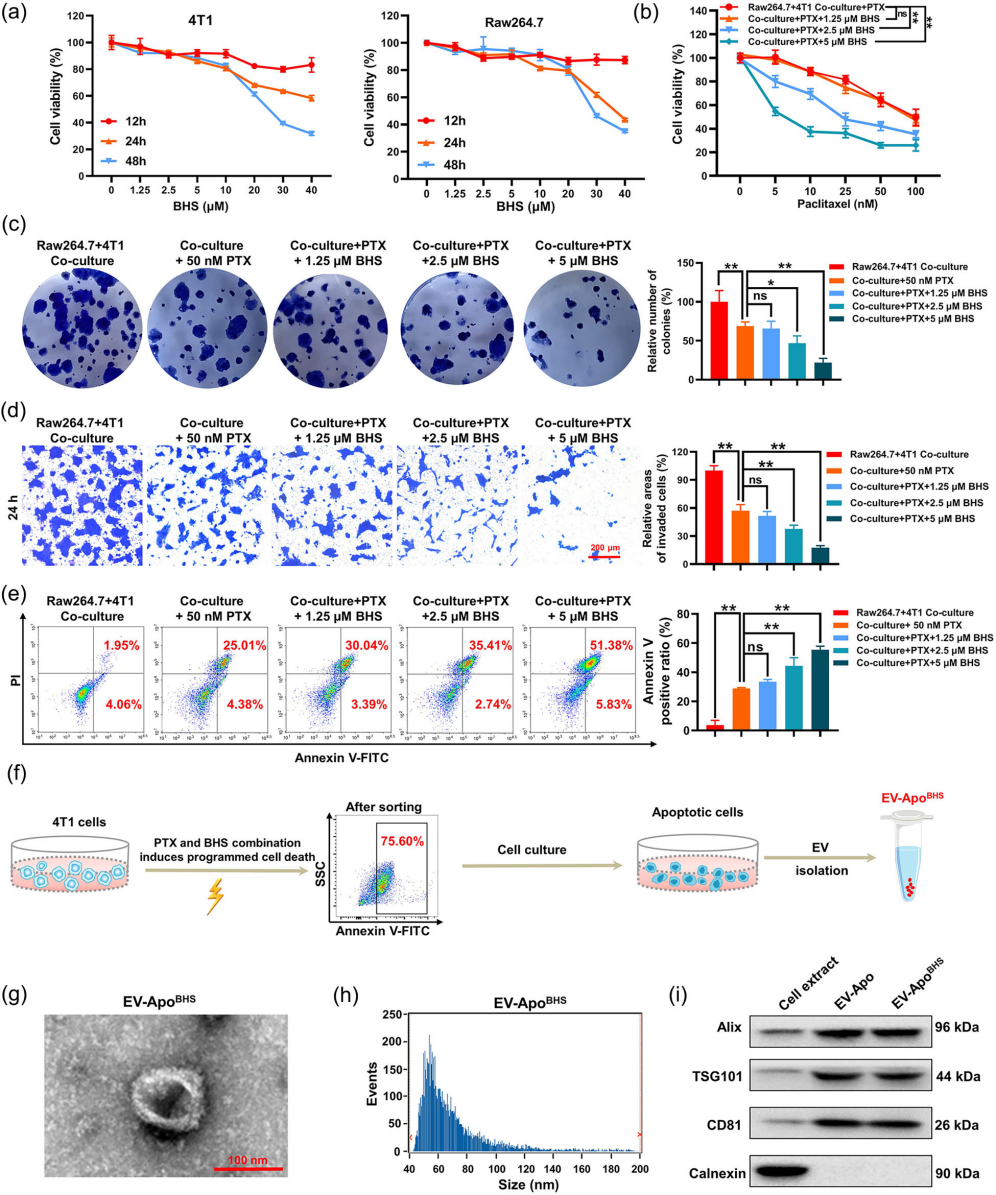
BHS synergizes with paclitaxel to suppress the invasion and chemoresistance of breast cancer cells co‐cultured with macrophages. (a) Cytotoxicity of BHS in 4T1 cells and Raw264.7 cells detected by the CCK8 assay. *n* = 3. (b) Cell viability of 4T1 cells in the co‐culture system after treatments with the combination of 1.25−5 µM BHS and 50 nM PTX determined with the CCK8 assay. *n* = 3. (c–e) BHS synergized with paclitaxel to suppress the colony formation, invasion, and apoptosis chemoresistance of the co‐cultured breast cancer cells. 4T1 cells were cultured alone or co‐cultured with Raw264.7 cells and treated as indicated for 24 or 48 h. *n* = 3. (f) A schematic diagram of the EV‐Apo^BHS^ isolation procedure. (g) A representative TEM image of EV‐Apo^BHS^. (h) Size distribution of EV‐Apo^BHS^ detected by a Flow NanoAnalyzer. (i) An immunoblot analysis of EV‐Apo^BHS^ detecting EV‐positive and negative biomarkers. *n* = 3. ^*^ *p* < 0.05, ^**^ *p* < 0.01.

Next, we aimed to investigate whether BHS synergized with paclitaxel to inhibit breast cancer invasion and chemoresistance. First, the CCK8 cytotoxic assay was performed on both TNBC cells, macrophages, and PBMCs to select non‐toxic concentrations of BHS. As shown in Figures 2(a) and S2, more than 90% of the above cells retained viability when treated with 1.25−5 µM BHS. Therefore, 1.25, 2.5 and 5 µM BHS were selected for the following in vitro assays. At concentrations of 2.5 and 5 µM, BHS significantly enhanced the effects of paclitaxel on suppressing the proliferation and colony formation abilities of 4T1 cells co‐cultured with macrophages (Figure 2b,c). More importantly, BHS at concentrations of 2.5 and 5 µM also synergized with paclitaxel to reduce the invasion and apoptosis resistance of 4T1 cells in the co‐culture system (Figure 2d,e). As stated above, paclitaxel‐elicited EV‐Apo could promote the invasion and chemoresistance of the co‐cultured breast cancer cells, whereas BHS at nontoxic concentrations synergized with paclitaxel to reverse that. Accordingly, we speculated that BHS may exert its synergistic effect with paclitaxel by modulating the EV‐Apo. To confirm this, EV‐Apo^BHS^ was isolated from the culture medium of apoptotic 4T1 cells co‐treated with BHS and paclitaxel (Figure 2f). The isolated EV‐Apo^BHS^ was also investigated by morphological observation, particle size determination (69.46 nm), and EV marker immunoblot assays (Figure 2g–i). Notably, both EV‐Apo and EV‐Apo^BHS^ exhibited an enrichment of the apoptotic marker Annexin V (Figure S3), which is consistent with their derivation from apoptotic cells.

**FIGURE 3:**
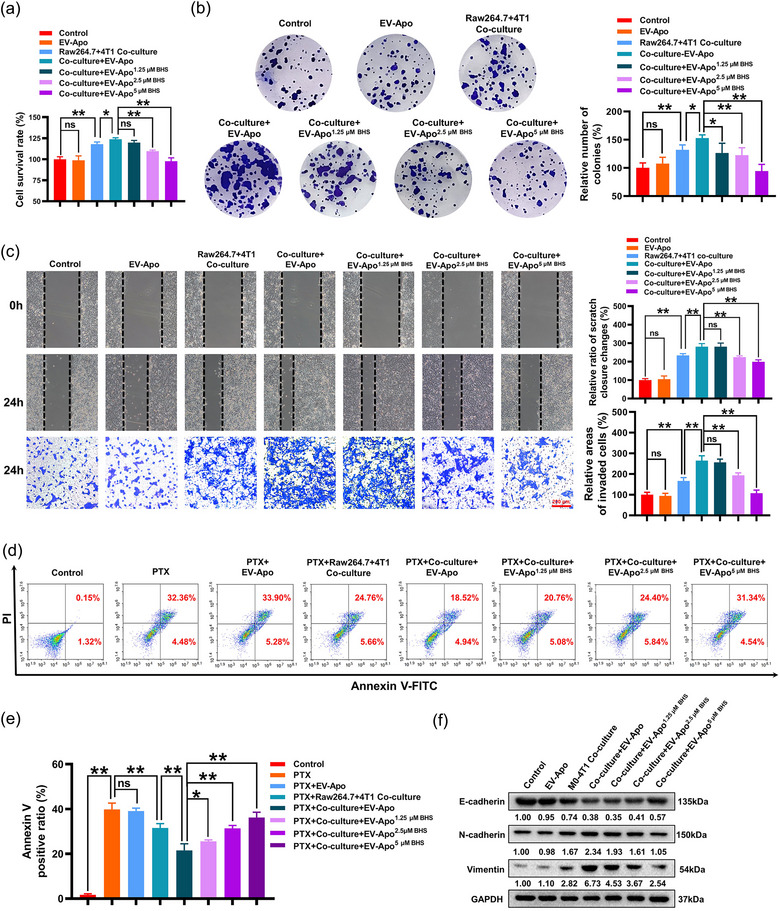
BHS chemosensitises breast cancer cells via modulating paclitaxel‐induced EV‐Apo signalling. (a, b) Proliferation (a) and colony formation (b) efficacies of 4T1 cells when treated as indicated for 48 h in the presence or absence of a Raw264.7 macrophage co‐culture. EVs were used at a concentration of 100 µg/mL. *n* = 3. (c) Migration and invasion efficacies of 4T1 cells when treated as indicated for 24 h. *n* = 3. (d–e) Apoptosis rates of 4T1 cells when treated as indicated for 48 h detected by Annexin V‐FITC/PI staining and flow cytometry. *n* = 3. (f) Expression levels of EMT‐related proteins in 4T1 cells when treated as indicated for 48 h. The concentration of paclitaxel was 50 nM. *n* = 3. ^*^ *p* < 0.05, ^**^ *p* < 0.01.

Next, the abilities of EV‐Apo and EV‐Apo^BHS^ to modulate the invasion and chemoresistance of 4T1 cells were compared. As shown in Figure 3(a,b), the proliferation and colony formation abilities of 4T1 cells in the macrophage co‐culture system were increased after treatment with 100 µg/mL EV‐Apo. However, EV‐Apo^2.5 µM BHS^ and EV‐Apo^5 µM BHS^ treatment inhibited the enhanced proliferation and colony formation activities stimulated by EV‐Apo. Similarly, EV‐Apo also increased the migration and invasion activities of the co‐cultured 4T1 cells. However, the co‐cultured 4T1 cells treated with EV‐Apo^2.5 µM BHS^ and EV‐Apo^5 µM BHS^ exhibited decreased migration and invasion activities compared to the EV‐Apo group (Figure 3c). In terms of apoptosis resistance, EV‐Apo administration also strongly decreased paclitaxel‐induced apoptosis of 4T1 cells co‐cultured with macrophages. However, EV‐Apo^2.5 µM BHS^ and EV‐Apo^5 µM BHS^ treatments recovered paclitaxel‐induced apoptosis of 4T1 cells in the co‐culture system (Figure 3d,e). Epithelial‐mesenchymal transformation (EMT) elevates the migration and invasion abilities of cancer cells (Qi et al., 2021). Compared to EV‐Apo, EV‐Apo^BHS^ demonstrated a diminished effect in inducing the EMT of 4T1 cells in the co‐culture system, as evidenced by the increased expression of E‐cadherin and decreased expression of N‐cadherin and vimentin (Figure 3f).

### 3.3 BHS inhibits the CXCL1 cargo in EV‐Apo and leads to decreased macrophage M2 polarization

**FIGURE 4:**
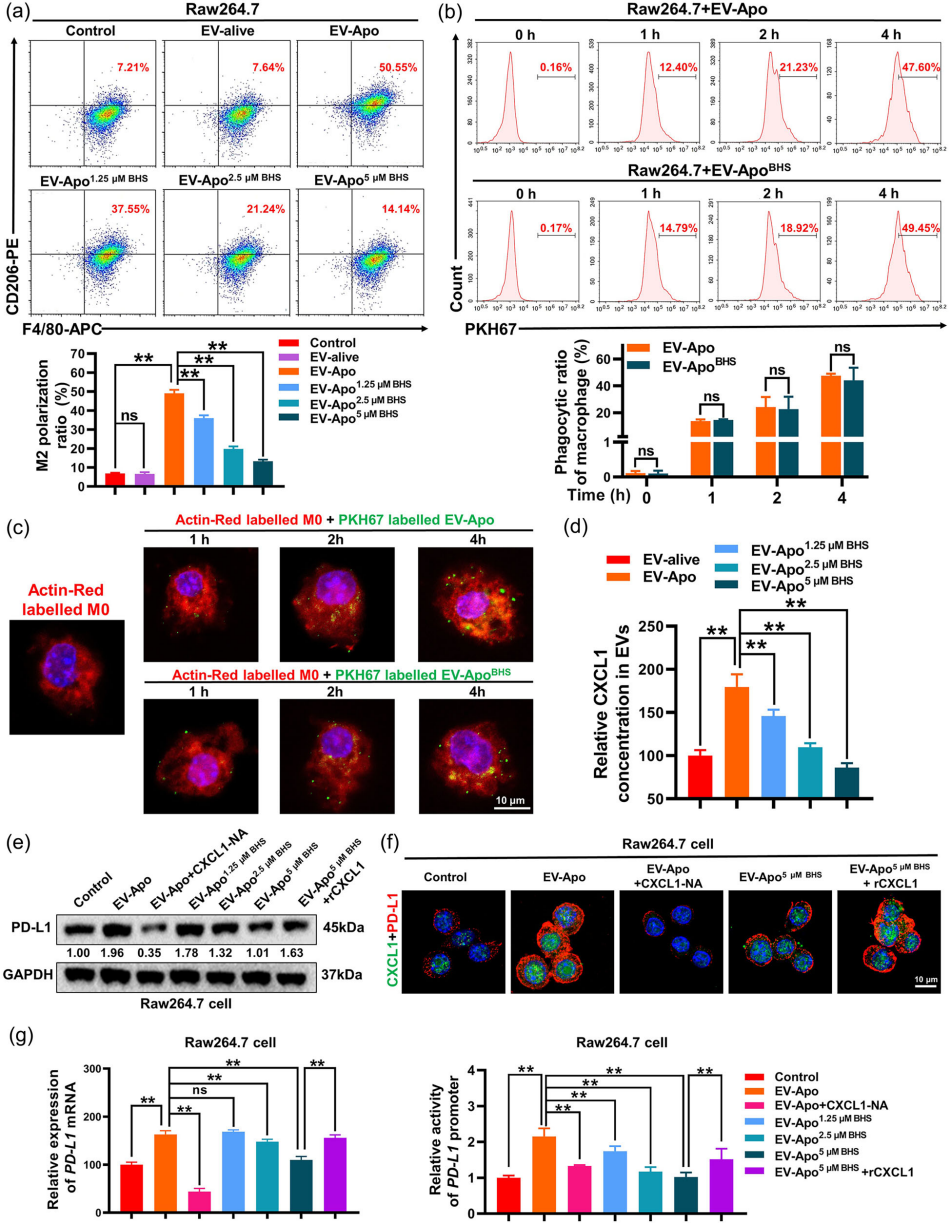
BHS inhibits the CXCL1 cargo in EV‐Apo and leads to decreased M2 polarization of Raw264.7 macrophages. (a) M2 polarization changes of Raw264.7 macrophages after treatments as indicated for 48 h were detected by an F4/80^+^/CD206^+^ population analysis. EVs were used at a concentration of 100 µg/mL. *n* = 3. (b) EV‐Apo and EV‐Apo^BHS^ uptake by Raw264.7 macrophages quantified by flow cytometry. EVs were labelled with PKH67 (green). *n* = 3. (c) EV‐Apo and EV‐Apo^BHS^ uptake by Raw264.7 macrophages visualized by immunofluorescence labelling. EVs were labelled with PKH67 (green), and Raw264.7 macrophages were labelled with Actin‐Red (red) and DAPI (blue). (d) CXCL1 concentrations in EV‐Apo and EV‐Apo^BHS^ detected by ELISA assay. *n* = 3. (e) Western blotting analysis of PD‐L1 expression in Raw264.7 cells when treated as indicated for 48 h. CXCL1‐NA concentration, 5 µg/mL. Recombinant CXCL1 (rCXCL1), 30 ng/mL. *n* = 3. (f) Immunofluorescence analysis of PD‐L1 expression in Raw264.7 cells when treated as indicated for 48 h. *n* = 3. (g) QPCR analysis of the mRNA expression levels (left) and promoter activity of *PD‐L1* (right) in Raw264.7 cells when treated as indicated for 48 h. *n* = 3. ^**^ *p* < 0.01.

**FIGURE 5:**
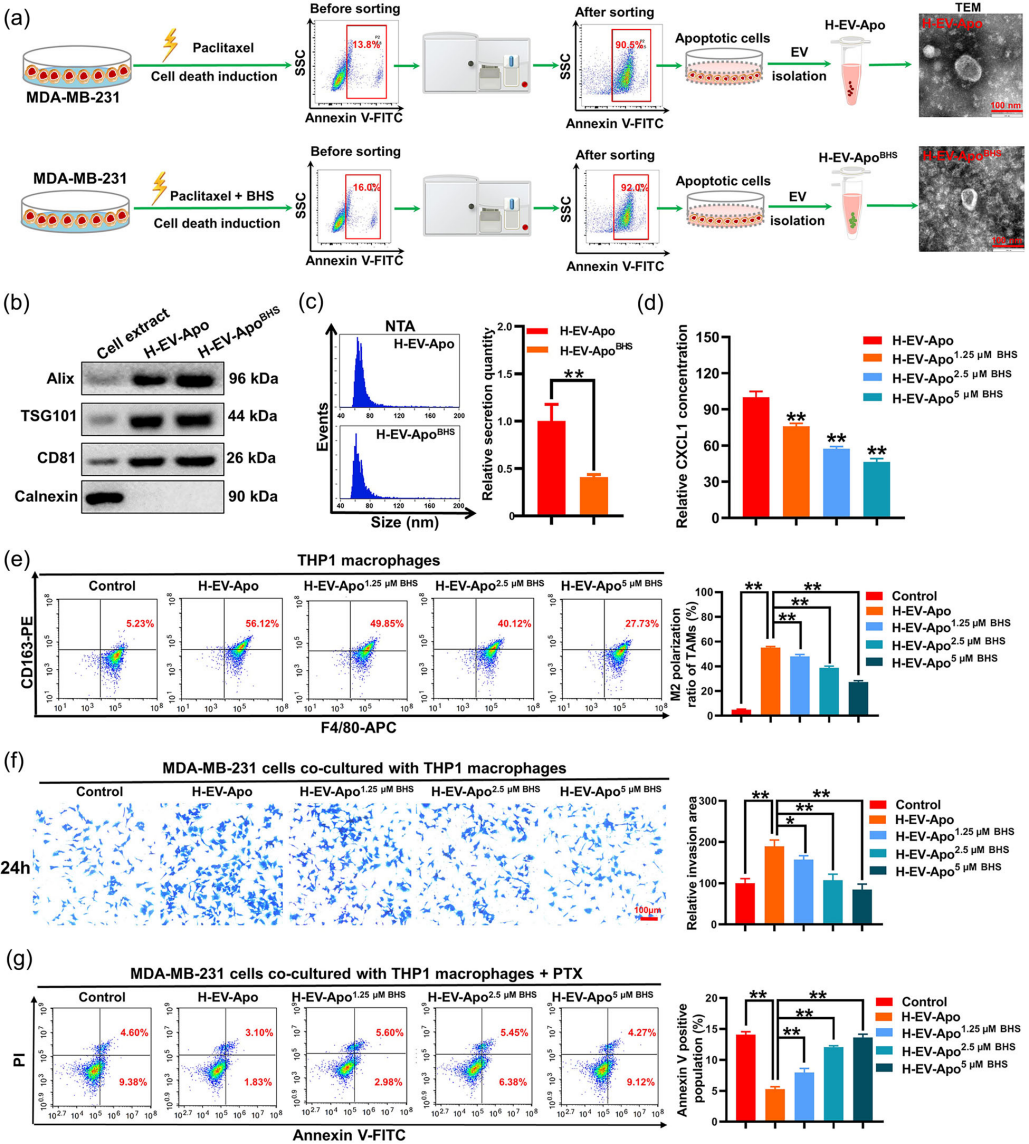
BHS inhibits the invasion and apoptosis resistance of the co‐cultured human MDA‐MB‐231 cells by suppressing EV‐Apo/CXCL1‐mediated M2 polarization of THP1 macrophages. (a) Diagram of the H‐EV‐Apo and H‐EV‐Apo^BHS^ separation procedures and their representative TEM images. Scale bar: 100 nm. (b) EV protein markers were identified by Western blotting. *n* = 3. (c) EV sizes and concentrations were detected by NTA analysis. *n* = 3. (d) CXCL1 concentrations in H‐EV‐Apo and H‐EV‐Apo^BHS^ were detected by ELISA assay. *n* = 3. (e) The polarization changes of THP1 macrophages after EV treatments (100 µg/mL) for 48 h. n = 3. (f) Invasion efficacy changes of the co‐cultured MDA‐MB‐231 cells after different EV treatments (100 µg/mL) as indicated for 24 h. *n* = 3. (g) Changes in apoptosis resistance of the co‐cultured MDA‐MB‐231 cells to paclitaxel (50 nM) after different EV treatments (100 µg/mL) for 48 h. *n* = 3. ^*^ *p* < 0.05, ^**^ *p* < 0.01.

Next, it was important to determine the underlying mechanisms accounting for the different bioactivities between EV‐Apo and EV‐Apo^BHS^. Macrophages are highly plastic cells, displaying different phenotypes and functions depending on exogenous stimuli. M2 phenotype macrophages usually facilitate breast cancer invasion and chemoresistance (Wang et al., 2021). Since both EV‐alive and EV‐Apo exhibited no direct effect on breast cancer cells, we speculated that the co‐cultured macrophages might be the key target of EVs. M2 phenotype macrophages express specific cellular markers on the surface, including the scavenger receptors CD206 and CD163 (Muller et al., 2022). As shown in Figure 4(a), 100 µg/mL EV‐Apo but not EV‐alive significantly induced the M2 polarization of Raw264.7 macrophages, characterized by elevated CD206 expression. In contrast, EV‐Apo^2.5 µM BHS^ and EV‐Apo^5 µM BHS^ achieved minimal effects on M2 polarization. Furthermore, the uptakes of PHK67‐labeled EV‐Apo and EV‐Apo^BHS^ by Raw264.7 macrophages were visualized and quantified by immunofluorescence and flow cytometry experiments (Figure 4b,c). Notably, both EV‐Apo and EV‐Apo^BHS^ can be uptaken by Raw264.7 macrophages in a time‐dependent manner. However, there were no significant differences in the uptake efficiencies between EV‐Apo and EV‐Apo^BHS^. Our previous study suggested that chemokine CXCL1 was the bioactive molecule in EV‐Apo and could activate downstream signalling PD‐L1 expression (Wang et al., 2024). In this study, knockdown of CXCL1 in EV‐Apo significantly inhibited EV‐Apo‐induced M2 polarization of Raw264.7 macrophages and therefore suppressed the invasion and apoptosis resistance of 4T1 cells co‐cultured with macrophages in vitro. (Figure S4). Therefore, we speculated that BHS treatment may inhibit CXCL1 cargo in EV‐Apo and finally lead to activity differences between EV‐Apo and EV‐Apo^BHS^. Consistent with this speculation, ELISA assay suggested that EV‐Apo^2.5 µM BHS^ and EV‐Apo^5 µM BHS^ loaded less CXCL1 than EV‐Apo (Figure 4d), leading to a decreased induction effect on PD‐L1 expression of Raw264.7 macrophages (Figures 4e–f and S5). Notably, BHS could reduce both the basal and chemotherapy‐induced CXCL1 expression and secretion into EVs, but not EV‐Apo alone (Figure S6). Next, mechanical explorations further suggested that EV‐Apo significantly elevated both the mRNA expression and promoter activity of *PD‐L1*, which could be partially abrogated by a CXCL1 neutralizing antibody (CXCL1‐NA). Similarly, EV‐Apo^2.5 µM BHS^ and EV‐Apo^5 µM BHS^ also limited the mRNA expression level and promoter activity of *PD‐L1* induced by EV‐Apo, but recombinant CXCL1 (rCXCL1) administration further reversed the effects of EV‐Apo^BHS^ (Figure 4g). To further validate the above findings, the human TNBC cell line MDA‐MB‐231 was utilized to isolate EV‐Apo. Similarly, the early apoptotic populations of MDA‐MB‐231 cells were sorted by flow cytometry. H‐EV‐Apo and H‐EV‐Apo^BHS^ were isolated from the supernatants of apoptotic human TNBC MDA‐MB‐231 cells induced by paclitaxel alone or a combination of paclitaxel and BHS, respectively. Multiple experiments including TEM observation, immunoblotting analysis of EV‐specific markers and NTA analysis confirmed the successful isolation of these two kinds of EVs (Figure 5a–c). It was found that the combination treatment with BHS significantly decreased the secretion number of H‐EV‐Apo^BHS^ (Figure 5c) and their CXCL1 cargo (Figure 5d). Furthermore, H‐EV‐Apo treatment also significantly induced the M2 polarization of human THP1 macrophages (Figure 5e), and therefore promoted the invasion and apoptosis resistance of MDA‐MB‐231 cells co‐cultured with THP1 macrophages (Figure 5f–g). Compared to H‐EV‐Apo, H‐EV‐Apo^BHS^ treatment exhibited decreased induction effects on the M2 polarization of THP1 macrophages, as well as the invasion and apoptosis resistance of the co‐cultured MDA‐MB‐231 cells. Altogether, these results indicate that BHS treatment decreases CXCL1 cargo in both mouse and human TNBC cell‐derived EV‐Apo, thereby suppressing EV‐Apo/CXCL1‐induced M2 macrophage polarization.

### 3.4 EV‐Apo is crucial for BHS to synergize with paclitaxel to suppress breast cancer chemoresistance and invasion

**FIGURE 6:**
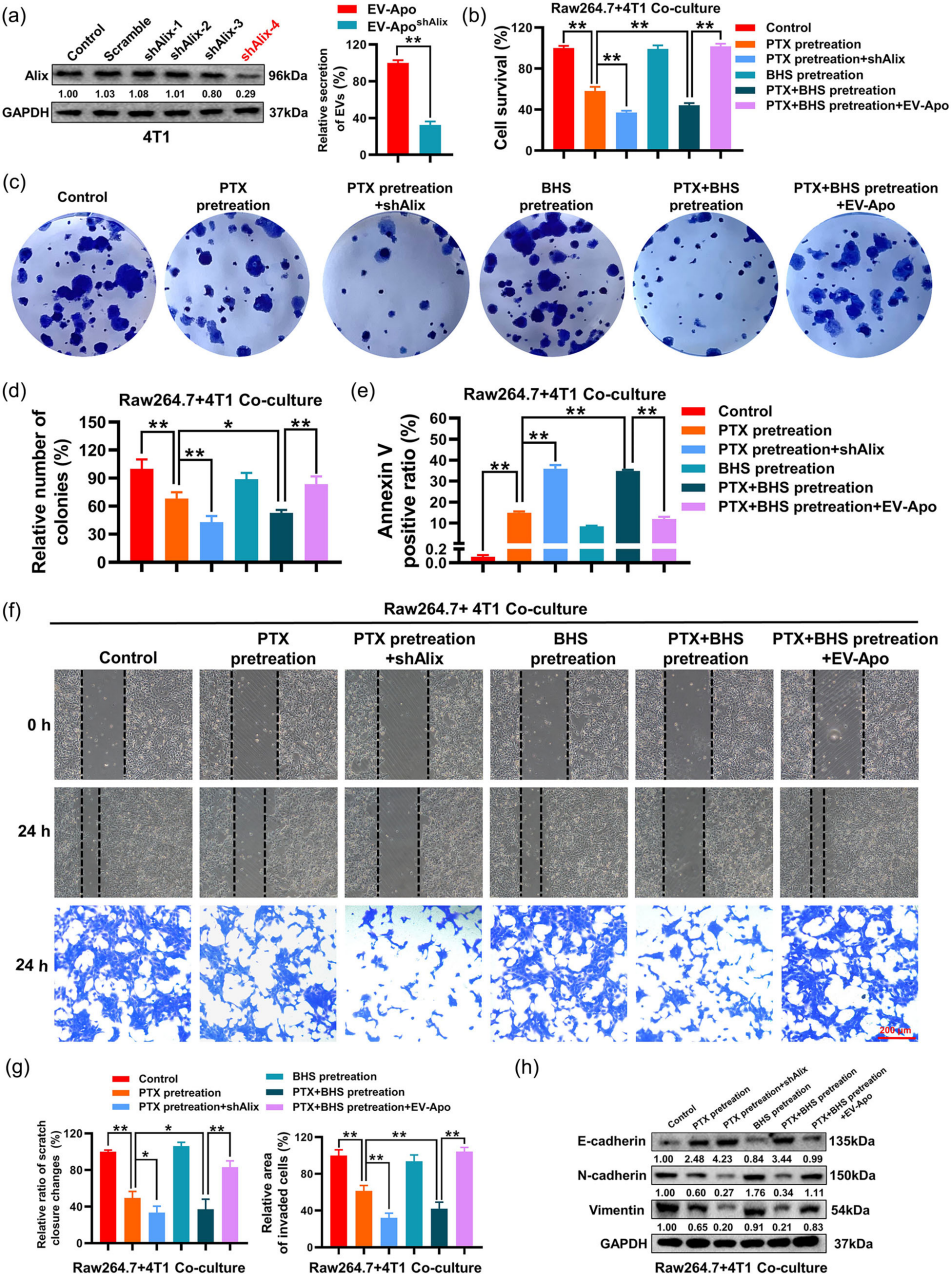
EV‐Apo is crucial for BHS to synergize with paclitaxel to suppress breast cancer chemoresistance and invasion. (a) Alix knockdown (left) significantly decreased the EV concentration (right) in the culture medium of 4T1 cells. The successful generation of 4T1/shAlix cells was validated by Western blotting. *n* = 3. (b–e) Alix knockdown significantly synergized with paclitaxel to inhibit the proliferation (b), colony formation (c–d), and apoptosis resistance (e) of breast cancer cells in the macrophage co‐culture system. 4T1 cells and 4T1/shAlix cells were pre‐treated with 5 µµ BHS, 50 nM paclitaxel or their combination for 48 h. *n* = 3. (f–g) Both Alix knockdown and BHS significantly synergized with paclitaxel to inhibit the migration and invasion of co‐cultured breast cancer cells. 4T1 cells and 4T1/shAlix cells were pre‐treated with BHS, paclitaxel or their combination for 24 h. *n* = 3. (h) The expression levels of EMT‐related proteins in 4T1 cells and 4T1/shAlix cells when the cells were pre‐treated as indicated for 48 h. *n* = 3. ^*^ *p* < 0.05, ^**^ *p* < 0.01.

Next, we aimed to investigate whether EV‐Apo signalling is crucial in mediating the synergistic effect of BHS with paclitaxel. Alix is one of the key modulators of exosome biogenesis and secretion. As expected, Alix knockdown significantly suppressed EV concentration in the culture medium of 4T1 cells (Figure 6a). Notably, Alix knockdown also significantly synergized with paclitaxel to inhibit the growth and apoptosis resistance of breast cancer cells in the macrophage co‐culture system. Similarly to the effect of Alix knockdown, BHS also significantly synergized with paclitaxel to inhibit the growth and apoptosis resistance of co‐cultured breast cancer cells, which could be partially reversed by the exogenous addition of EV‐Apo (Figure 6b–e). Additionally, both Alix knockdown and BHS significantly synergized with paclitaxel to inhibit the migration, invasion and EMT of the co‐cultured breast cancer cells, whereas the synergistic effect of BHS was partially abrogated by the exogenous addition of EV‐Apo (Figure 6f–h). Notably, EV‐Apo addition partially abrogated the effects of Alix knockdown on paclitaxel‐induced apoptotic death and inhibition of migration/invasion of 4T1 cells (Figure S7). These results suggest that EV‐Apo signalling plays a crucial role in mediating the synergistic effect of BHS with paclitaxel in breast cancer.

### 3.5 BHS inhibits EV‐Apo signalling by suppressing ILV biogenesis via CXCL1

**FIGURE 7:**
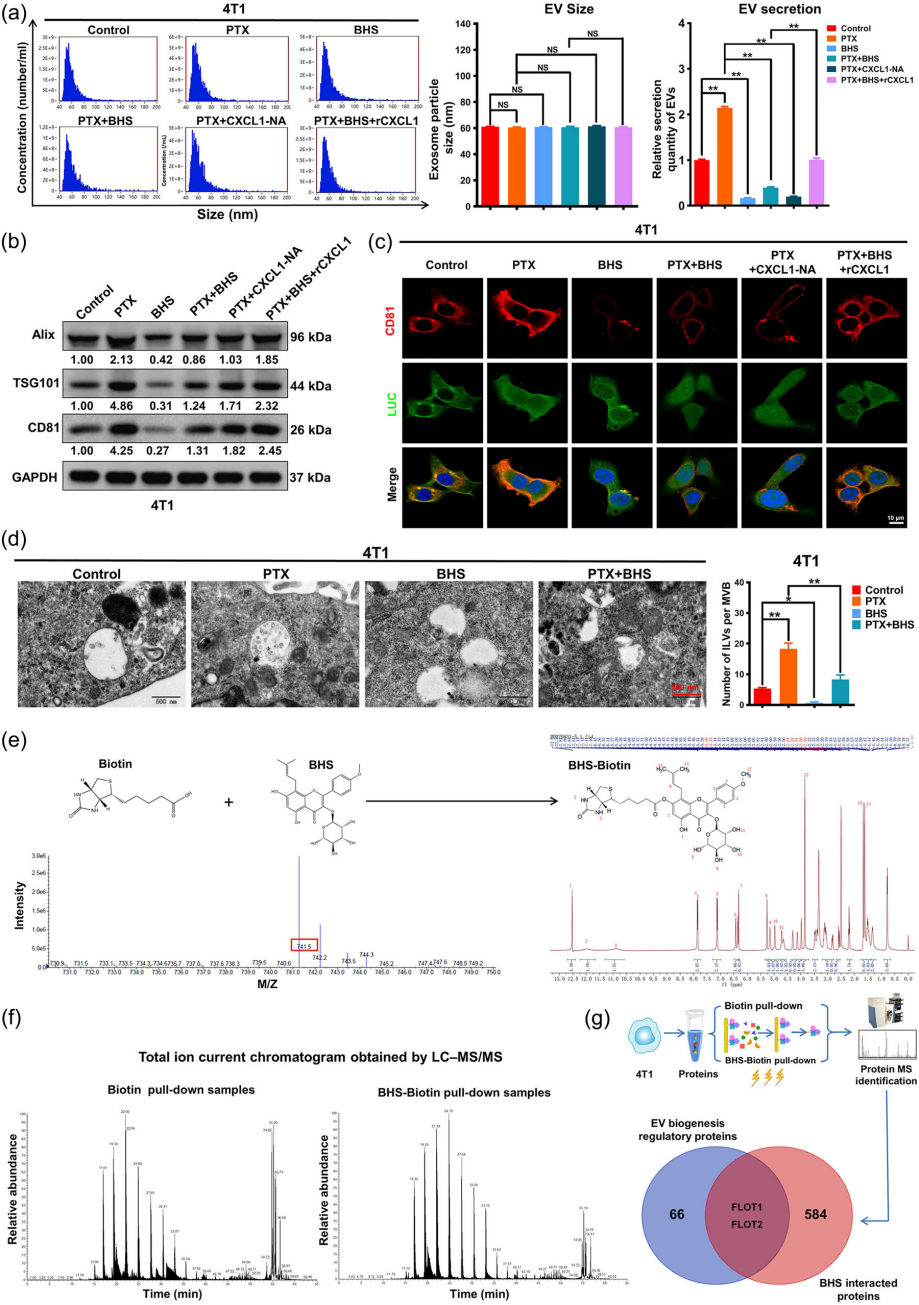
BHS inhibits EV‐Apo signalling by suppressing ILV biogenesis via CXCL1. (a) The size distribution and concentrations of different EVs isolated from the culture medium of 4T1 cells after treatments as indicated for 48 h. PTX, 50 nM; BHS, 5 µM; CXCL1‐NA, 5 µg/mL; rCXCL1, 30 ng/mL. *n* = 3. (b) Western blotting assay of the expression levels of EV markers in 4T1 cells after treatments as indicated for 48 h. *n* = 3. (c) Immunofluorescence analysis of CD81 protein expression level in 4T1‐Luc cells when treated as indicated for 48 h. *n* = 3. (d) TEM analysis of ILVs in MVBs of 4T1 cells when treated with 50 nM PTX, 5 µM BHS or their combination for 48 h. *n* = 3. (e) Biotin‐tagged BHS was synthesized chemically and its successful synthesis was confirmed by the mass spectrum (left) and 1H NMR spectrum (right). (f) A total ion current chromatogram of the pull‐down samples obtained by LC‐MS/MS. (g) FLOT1 and FLOT2 were selected by intersecting BHS‐interacted proteins with EV biogenesis regulatory proteins. ^*^ *p* < 0.05, ^**^ *p* < 0.01.

Since EV‐Apo is crucial for the synergistic effect between BHS and paclitaxel to suppress breast cancer, we set out to determine whether BHS could decrease the secretion and biogenesis of EV‐Apo. As shown in Figure 7(a), although both paclitaxel and BHS exhibited a minimal effect on the sizes of the secreted EVs, paclitaxel treatment strongly elevated EV‐Apo secretion from 4T1 cells, whereas BHS treatment remarkably decreased that. Similar to the effect of CXCL1‐NA, BHS also dramatically inhibited paclitaxel‐induced EV‐Apo secretion, which could be partially abrogated by the exogenous addition of CXCL1. These results indicated that BHS decreased paclitaxel‐induced EV‐Apo secretion by modulating the CXCL1 pathway. Additionally, both immunoblot and immunofluorescence analyses showed that BHS attenuated the expression levels of both the basal and paclitaxel‐induced exosomal markers; this effect could be partially abrogated by exogenous CXCL1 addition (Figure 7b,c), suggesting that BHS may decrease the biogenesis of EV‐Apo by downregulating the CXCL1 pathway. Exosomes are essentially ILVs generated by the inward budding of endosomal MVBs (Song et al., 2019). A TEM analysis showed that paclitaxel treatment significantly increased the number of ILVs per MVB, whereas BHS significantly decreased that. Notably, BHS significantly suppressed paclitaxel‐induced ILV elevation per MVB (Figure 7d). These results clearly showed that BHS suppressed paclitaxel‐induced ILV biogenesis. To determine the molecular target via which BHS inhibited ILV biogenesis, biotinylated‐BHS (BHS‐Biotin, molecular weight 740.8) was chemically synthesized and validated by the mass spectrum (*m*/*z* = 741.5) and 1H NMR spectrum (Figure 7e). Pull‐down‐MS was conducted using BHS‐Biotin to identify the direct binding protein of BHS in 4T1 cell protein lysates. Silver staining of the pull‐down samples suggested that there were significant differences between biotin‐pull‐down samples and BHS‐Biotin pull‐down samples. An MS analysis identified 584 differentially expressed proteins (DEPs) between the biotin and BHS‐Biotin groups. By intersecting with EV biogenesis regulatory proteins, two proteins named flotillin 1 (FLOT1) and flotillin 2 (FLOT2) were implicated, and FLOT2 had higher folds than FLOT1 (4.47 vs. 3.87 folds) (Figures 7f,g and S8).

### 3.6 BHS decreases ILV biogenesis by directly inhibiting the FLOT2/RAB31 interaction

**FIGURE 8:**
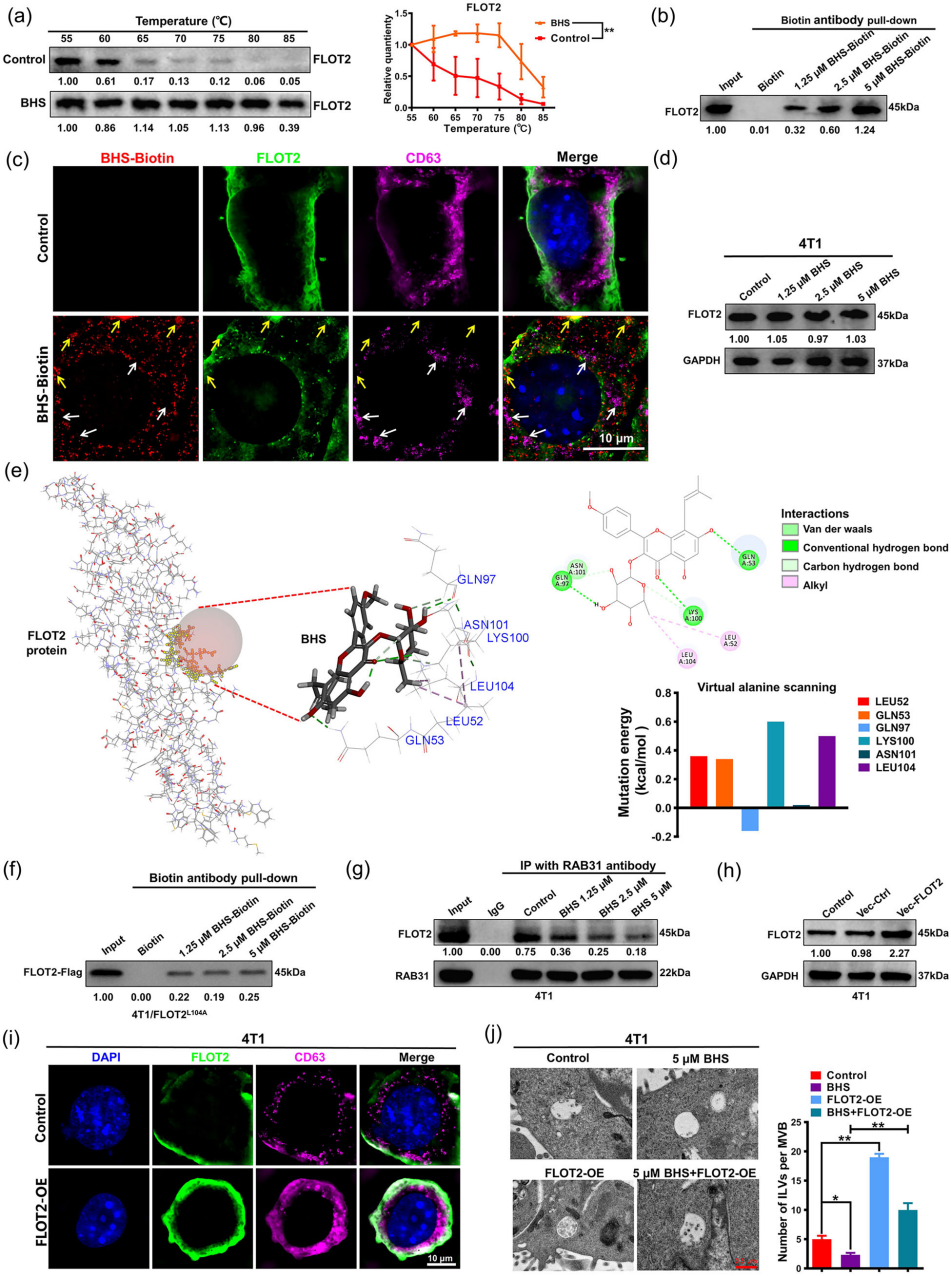
BHS decreases ILV biogenesis by directly inhibiting the FLOT2/RAB31 interaction. (a) A CETSA analysis of the thermal stability of FLOT2 in 4T1 cells. *n* = 3. (b) A Co‐IP analysis of the interaction between 1.25 and 5 µM BHS and FLOT2 in 4T1 cells. *n* = 3. (c) Immunofluorescence analysis and ultra‐high‐resolution microscopy observation of FLOT2 and CD63 expression levels in 4T cells when treated with 5 µM BHS‐Biotin for 48 h. *n* = 3. Yellow arrows indicate high distribution regions and colocalization regions of BHS‐Biotin and FLOT2, whereas CD63 exhibits low expression in these regions. White arrows indicate low BHS‐Biotin distribution regions, whereas CD63 exhibits high expression in these regions. (d) Western blotting analysis of FLOT2 expression levels in 4T cells when treated with 1.25−5 µµ BHS for 48 h. *n* = 3. (e) Computer‐aided virtual docking between BHS and FLOT2 (left) and subsequent virtual alanine scanning (right) were conducted to predict the potential binding between BHS and the ^104^LQTL^107^ motif of the FLOT2 protein. (f) A Co‐IP analysis of the interaction between 1.25 and 5 µM BHS‐Biotin and Flag‐tagged FLOT2^L104A^ in 4T1/FLOT2^L104A^ mutation cells. *n* = 3. (g) A Co‐IP analysis of the interaction activity between RAB31 and FLOT2 in 4T1 cells when treated as indicated for 48 h. *n* = 3. (h) Successful generation of 4T1/FLOT2‐OE cells validated by Western blotting. n = 3. (i) An immunofluorescence analysis of CD63 and FLOT2 protein expression levels in 4T1 and 4T1/FLOT2‐OE cells. *n* = 3. (j) A TEM analysis of ILVs per MVBs of 4T1 cells and 4T1/FLOT2‐OE cells when treated with 5 µM BHS for 48 h. *n* = 3. ^*^ *p* < 0.05, ^**^ *p* < 0.01.

Next, it was essential to identify whether FLOT2 is the critical target of BHS in inhibiting EV‐Apo biogenesis as well as the underlying mechanisms. As shown in Figure 8(a), a CETSA analysis showed that BHS significantly elevated the thermal stability of FLOT2, suggesting there may be an interaction between BHS and FLOT2. Furthermore, the Co‐IP assay further confirmed the concentration‐dependent interaction between BHS and FLOT2 (Figure 8b). Moreover, immunofluorescence analysis using ultra‐high‐resolution microscopy indicated the colocalization between BHS and FLOT2, where the expression of the exosome marker CD63 was significantly decreased (Figure 8c). Notably, immunoblot analysis showed that BHS at concentrations of 1.25−5 µµ exhibited little effect on FLOT2 expression (Figure 8d). These results suggest that BHS may inhibit FLOT2‐mediated EV biogenesis by direct binding. Therefore, an *in‐silico* docking simulation was performed to investigate the binding mode between BHS and FLOT2. It has been reported that the RAB31‐FLOT2 machinery induces the budding of the MVB membrane to form ILVs (Wei et al., 2021). Since the ^104^LQTL^107^ motif in the SPFH domain of FLOT2 is responsible for its interaction with RAB31 (Wei et al., 2021), the binding site between BHS and FLOT2 was set on the ^104^LQTL^107^ motif. After performing a flexible docking procedure, BHS was successfully docked into the FLOT2 protein, and there was a potential alkyl interaction between BHS and the LEU104 residue of the FLOT2 protein. A virtual alanine mutation scanning was subsequently conducted to pinpoint pivotal residues influencing the binding affinity between BHS and FLOT2. A noteworthy increase in mutation energy was observed upon substituting LEU104 of FLOT2 with alanine (Figure 8e). As stated above, BHS exhibited a concentration‐dependent binding affinity with FLOT2 in 4T1 cells. However, after the transfection of Flag‐tagged FLOT2^L104A^ mutant into 4T1 cells, the binding affinity between BHS and FLOT2^L104A^‐Flag was remarkably decreased and remained relatively unchanged despite escalating BHS concentrations in 4T1/FLOT2^L104A^ mutant cells (Figure 8f). These results suggested that LEU^104^ may play a crucial role in the binding of BHS to FLOT2. Based on the existing reports and our results, we speculated that BHS may bind with FLOT2 and therefore interrupt the formation of the RAB31‐FLOT2 machinery, and finally lead to ILV biogenesis blockage. Since the FLOT2^L104A^ mutant retained binding to RAB31 (Figure S9), 4T1 cells with wild‐type FLOT2 were chosen to validate this speculation. As expected, the Co‐IP assay showed that 1.25−5 µµ BHS treatment inhibited the interaction activity between RAB31 and FLOT2 in 4T1 cells in a dose‐dependent manner (Figure 8g). More importantly, FLOT2 overexpression significantly abrogated the inhibitory effect of BHS on ILV biogenesis in 4T1 cells, characterized by decreased expression of the EV marker CD63 and decreased number of ILVs per MVB (Figure 8h–j). To sum up, BHS decreases ILV biogenesis by inhibiting the FLOT2/RAB31 interaction.

### 3.7 BHS chemosensitises breast cancer in vivo by suppressing EV‐Apo/TAM/PD‐L1 signalling

**FIGURE 9:**
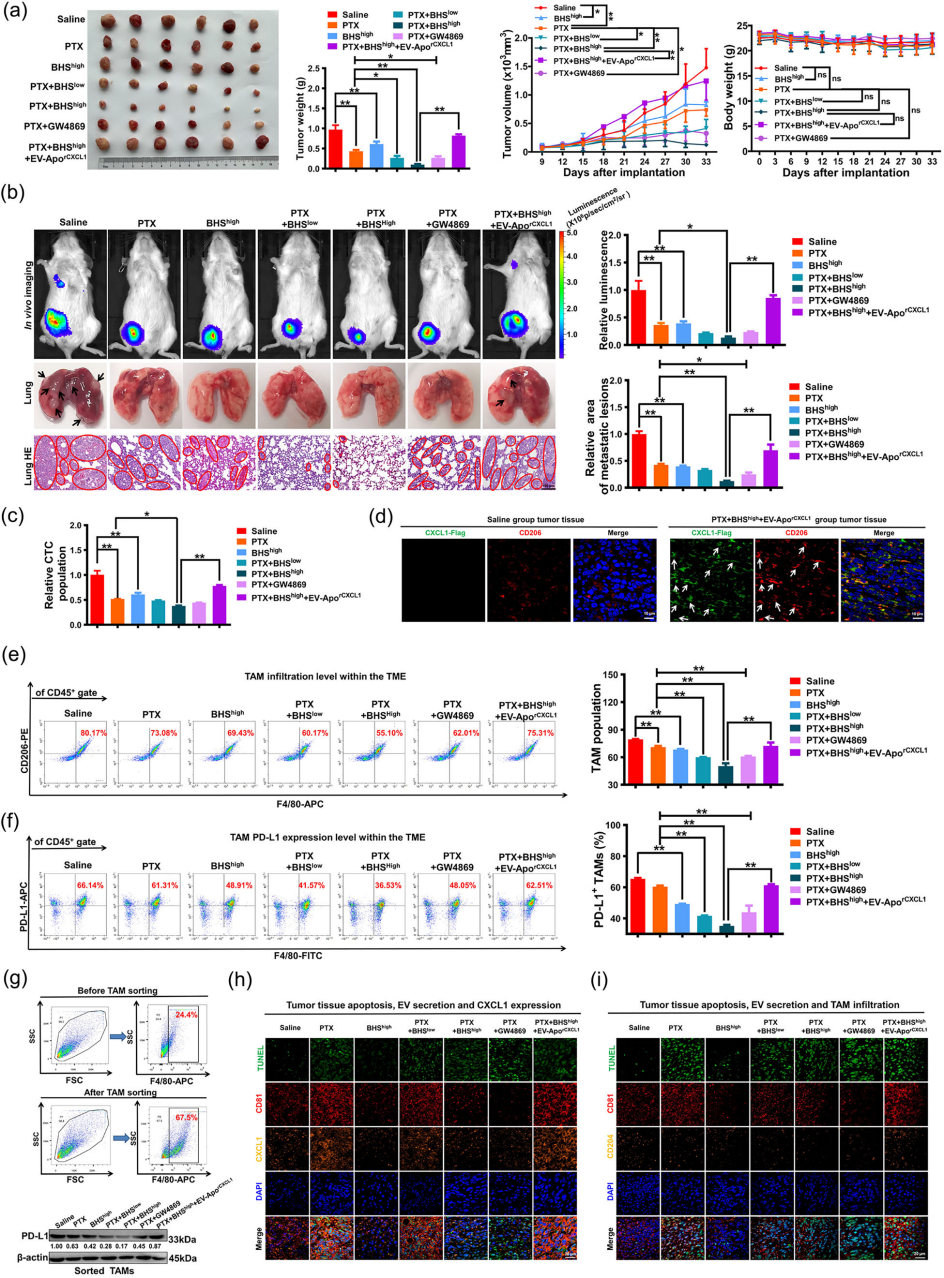
BHS chemosensitises breast cancer in immunodeficient NSG mice by suppressing EV‐Apo/TAM/PD‐L1 signalling. (a) Representative images of tumours as well as the statistical analysis of tumour weights, tumour volumes and body weights of NSG mice among different groups. The immunodeficient NSG mice lack B, T, and natural killer (NK) cells. Paclitaxel (10 mg/kg/3d), BHS (10 or 20 mg/kg/d), and GW4869 (1.25 mg/kg/d) were administrated intraperitoneally, whereas EV‐Apo^rCXCL1‐Flag^ (200 µg/kg/3d) was administrated by a peritumoral injection. *n* = 6. (b) Representative images of the in vivo imaging assay, the harvested lungs and the lung HE staining assay; *n* = 3. (c) The CTCs in the peripheral blood of mice among different groups were detected by QPCR. *n* = 3. (d) An immunofluorescence analysis of tumour tissues suggested that the Flag‐tagged EV‐Apo/CXCL1 signal (green) was predominantly phagocytosed by CD206^+^ TAMs (red). White arrows indicate the colocalization regions of CXCL1 and CD206. *n* = 3. (e, f) Infiltration levels of CD45^+^/F4/80^+^/CD206^+^ TAMs and CD45^+^/F4/80^+^/PD‐L1^+^ TAMs in the TME of mice among different groups. *n* = 3. (g) Immunoblot analysis of PD‐L1 expression (*n* = 3) in sorted TAMs from breast tumours in each group. (h) TUNEL staining of tissue apoptosis in breast tumour tissues and an immunofluorescence analysis of CD81, CXCL1 and CD204 expression levels in breast tumour tissues of different groups. ^*^ *p* < 0.05, ^**^ *p* < 0.01.

Finally, we investigated whether BHS synergized with paclitaxel to inhibit the growth and metastasis of breast tumours in vivo. As stated above, paclitaxel elicited pro‐metastatic EV‐Apo that induced macrophage M2 polarization to promote the invasion and apoptosis resistance of the co‐cultured breast cancer cells. To exclude the influence of other immune cells, immunodeficient NSG mice lacking B, T, and natural killer (NK) cells were used. Luciferase‐expressing 4T1‐Luc cells were implanted into the mammary fat pads of female NSG mice to generate an orthotopic model for fluorescence tracking of breast tumour growth and metastasis. As shown in Figure 9(a–c), the combination of paclitaxel (10 mg/kg/3d) and BHS (20 mg/kg/d) achieved a significant synergistic effect on inhibiting the growth and lung metastasis of breast cancer, characterized by the decreased tumour weights, volumes, fluorescence intensities, lung metastatic lesion areas, and peripheral blood CTC counts. BHS and paclitaxel combination did not bring additional toxic side effects, since no significant impact on mouse weight was observed in the BHS and paclitaxel combination groups. Furthermore, the exosome secretion inhibitor GW4869 (1.25 mg/kg/d) also significantly synergized with paclitaxel to inhibit the growth and lung metastasis of breast tumours. Moreover, the exogenous addition of CXCL1‐overexpressing EV‐Apo (EV‐Apo^rCXCL1^, 200 µg/20 g, q3d) strongly reversed the synergistic inhibition effect between BHS and paclitaxel on breast cancer growth and lung metastasis. These results suggested that BHS may synergize with paclitaxel to suppress breast cancer by suppressing the EV‐Apo/CXCL1 pathway. Significantly, immunofluorescence analysis further suggested that the Flag‐tagged EV‐Apo/CXCL1 signal was predominantly phagocytosed by CD206^+^ TAMs in the TME of the immunodeficient NSG mice (Figure 9d). In fact, our previous study has confirmed that the EV‐Apo/CXCL1 signal was predominantly phagocytosed by CD206^+^ TAMs within the TME of immunocompetent Balb/c mice, despite the presence of other immune cells (Wang et al., 2024). More importantly, similarly to the effect of the GW4869 (exosome secretion inhibitor) and paclitaxel combination, BHS and paclitaxel combination also significantly decreased the infiltration of TAMs and attenuated their PD‐L1 expression, whereas the exogenous addition of CXCL1‐overexpressing EV‐Apo dramatically reversed that (Figure 9e–f). An immunoblot analysis of PD‐L1 expression in the sorted TAMs from breast tumours in each group further confirmed this result (Figure 9g). Additionally, immunofluorescence analysis of breast tumours showed that BHS synergized with paclitaxel to significantly decrease the expression levels of CD81, CXCL1, and CD204, suggesting that BHS suppressed paclitaxel‐induced EV‐Apo biogenesis, CXCL1 secretion, and TAM infiltration (Figure 9h–i). Notably, a decrease in CXCL1 expression level was observed in the tumour tissue of the GW4869 treatment group. Considering that CXCL1 is among the most abundantly secreted chemokines by TAMs (Wang et al., 2020), this phenomenon may arise from the suppression of macrophage infiltration in tumours, given the limited influence of GW4869 treatment on CXCL1 secretion by breast cancer 4T1 cells (Figure S10).

**FIGURE 10:**
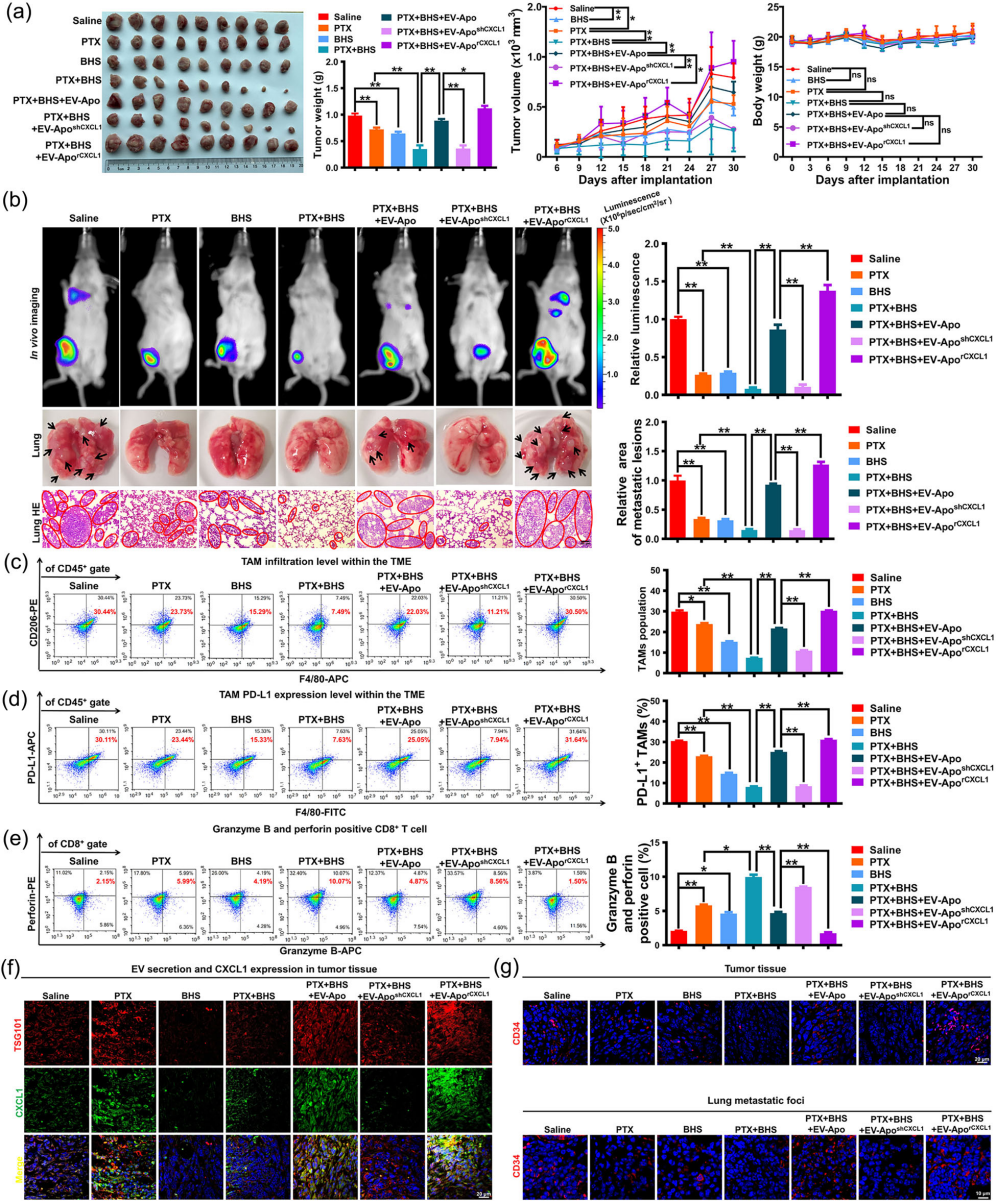
BHS chemosensitises breast cancer in immunocompetent Balb/c mice by suppressing EV‐Apo/TAM/PD‐L1 signalling. (a) Representative images of tumours as well as the statistical analysis of tumour weights, tumour volumes and body weights of Balb/c mice among different groups. Paclitaxel (10 mg/kg/3d) and BHS (20 mg/kg/d) were administrated intraperitoneally, whereas EVs (200 µg/kg/3d) were administrated by a peritumoral injection. *n* = 9. (b) Representative images of the in vivo imaging assay, the harvested lungs and the lung HE staining assay. *n* = 3. (c, d) Infiltration levels of CD45^+^/F4/80^+^/CD206^+^ TAMs and CD45^+^/F4/80^+^/PD‐L1^+^ TAMs in the TME of mice among different groups. *n* = 3. (e) Perforin/granzyme B production activity of cytotoxic CD8^+^ T cells in the TME of mice among different groups. *n* = 3. (f) Immunofluorescence analysis of TSG101 (EV‐specific marker) and CXCL1 expression levels in breast tumour tissues of different groups. (g) Immunofluorescence analysis of CD34 (vascular marker) in breast tumour tissues and lung metastatic foci of different groups. ^*^ *p* < 0.05, ^**^ *p* < 0.01.

4T1 cells were originally obtained from spontaneous breast cancer in Balb/c mice. Therefore, syngeneic Balb/c mice with intact immune systems were subsequently utilized to determine whether BHS can chemosensitise breast cancer in immunocompetent mice by inhibiting the EV‐Apo/TAM/PD‐L1 pathway. As shown in Figure 10, it was found that the combination of paclitaxel (10 mg/kg/3d) and BHS (20 mg/kg/d) exerted a significant synergistic effect on inhibiting the growth and lung metastasis of 4T1‐Luc breast cancer in Balb/c mice, as evidenced by the decreased tumour weights, volumes, fluorescence intensities, and lung metastatic lesion areas. Furthermore, the exogenous addition of EV‐Apo significantly reversed the synergistic inhibition effect between BHS and paclitaxel on breast cancer growth and lung metastasis, and this effect could be partially mitigated or further enhanced by knocking down or overexpressing CXCL1 in EV‐Apo, respectively (Figure 10a,b). These results indicate that BHS may synergize with paclitaxel to suppress breast cancer by suppressing the EV‐Apo/CXCL1 pathway. Moreover, the combination of BHS and paclitaxel also markedly decreased the infiltration of TAMs and attenuated their PD‐L1 expression by suppressing the EV‐Apo/CXCL1 pathway, consequently enhancing the perforin/granzyme B activity in CD8^+^ T cells (Figure 10c–e). Additionally, immunofluorescence analysis of breast tumours revealed that BHS synergized with paclitaxel to significantly decrease the expression of TSG101 and CXCL1, indicating that paclitaxel‐induced EV‐Apo biogenesis and CXCL1 secretion were suppressed (Figure 10f). Altogether, BHS synergizes with paclitaxel to inhibit breast cancer growth and lung metastasis by suppressing EV‐Apo/CXCL1‐induced TAM infiltration and PD‐L1 expression in an immunocompetent mouse model. Besides remodelling the TME, the effect of BHS on neoangiogenesis and autophagic activity within tumours and lung metastatic foci were also investigated. As shown in Figure 10(g), tissue immunofluorescence was employed to detect the expression of the vascular marker CD34 in tumour tissues and lung metastatic foci. The results revealed that BHS, when combined with paclitaxel, synergistically suppressed neoangiogenesis in both tumour tissues and lung metastatic foci, which was also closely correlated with EV‐Apo/CXCL1. Additionally, as shown in Figure S11, primary tumour cells from both tumour tissues and pulmonary metastases were isolated, and mRFP‐GFP‐LC3 adenoviral vectors were transfected to mark the autolysosomes and autophagosomes. The findings suggested that BHS inhibited the autophagic activity induced by paclitaxel in tumour tissues and lung metastases, and EV‐Apo/CXCL1 signalling also significantly contributed to this process. Collectively, EV‐Apo/CXCL1 signalling appears to play a crucial role in multiple tumour‐associated biological processes, thereby acting its potential as a valuable therapeutic target for cancer treatment. A systematic investigation into its diverse biological functions is imperative in future studies.

## 4 DISCUSSION

**FIGURE 11:**
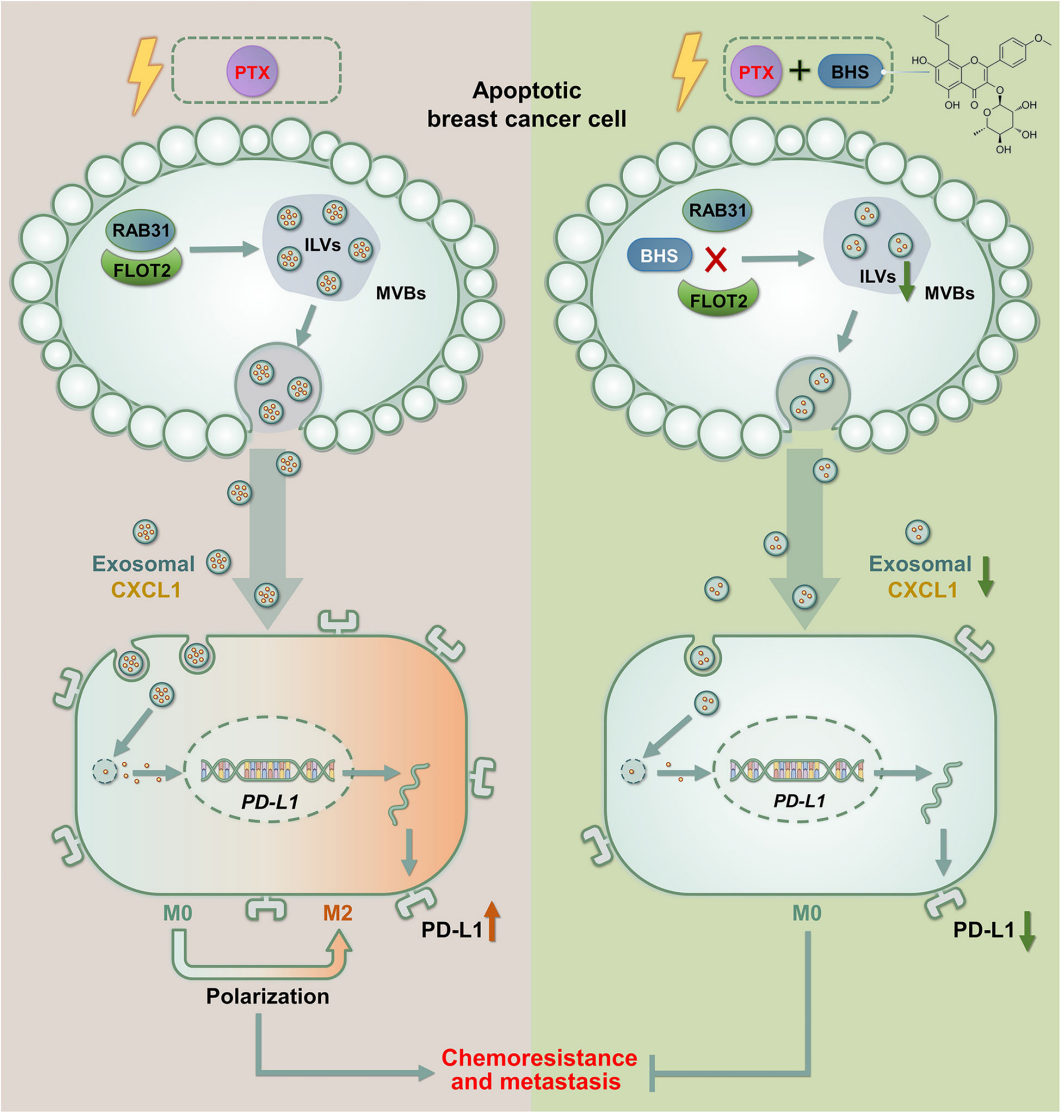
BHS inhibits breast cancer chemoresistance and metastasis by suppressing EV‐Apo/CXCL1 biogenesis and secretion.

TNBC is the most aggressive and metastatic subtype of breast cancer, and chemotherapy remains the mainstream systemic treatment option available for patients with TNBC. Unfortunately, accumulating evidence has suggested that cytotoxic chemotherapy may itself accelerate cancer resistance and metastasis while decreasing the primary tumour burden (Karagiannis et al., 2017; Wills et al., 2021). The involved mechanisms include CSC enrichment, autophagy induction, EMT enhancement, neoangiogenesis, and TME or PMN remodelling by inducing inflammation and cytokine production. For example, George *et al.* reported that paclitaxel neoadjuvant chemotherapy may delay the growth of breast cancer but accelerate its metastatic dissemination by remodelling a pro‐metastatic TME (Karagiannis et al., 2017). Notably, most previous studies have primarily concentrated on assessing the direct impact of chemotherapy on the biological behaviour of cancer cells or other cellular constituents within the TME. Recently, emerging evidence has suggested that EVs that are produced in response to chemotherapy could induce cancer chemoresistance and metastasis (Keklikoglou et al., 2019; Wills et al., 2021). However, the precise molecular mechanisms and the corresponding pharmacological intervention strategies are poorly illustrated. Herein, we demonstrated that paclitaxel chemotherapy elicited CXCL1‐enriched EV‐Apo from apoptotic breast cancer cells, which promoted the chemoresistance and metastasis of breast cancer by polarizing M2 macrophages through activating PD‐L1 signalling. More importantly, BHS, a highly effective natural product with low toxicity, was presented as a promising chemosensitiser to inhibit breast cancer chemoresistance and metastasis by suppressing EV‐Apo/CXCL1 biogenesis and secretion (Figure 11). Although several studies have demonstrated that chemotherapy elicited pro‐metastatic EVs in breast cancer (Keklikoglou et al., 2019; Wills et al., 2021; Yang et al., 2021), to our knowledge, this pioneering study focused on chemotherapy‐induced EVs secreted from apoptotic cancer cells and identified EV‐Apo/CXCL1/TAMs as a novel immune escape axis to fuel chemotherapy‐induced resistance and metastasis. Additionally, for the first time, we reported the novel pharmacological function of BHS to modulate both the biogenesis and cargo of EV‐Apo and identified FLOT2 as its direct target. These results identify EV‐Apo as a new approach for investigating chemotherapy‐induced resistance and metastasis in addition to the chemosensitising mechanisms of BHS.

Recently, EVs have shown great potential in the diagnosis and treatment of malignancies. Numerous studies have suggested that EVs released from both cancer cells and stromal cells could promote the chemoresistance and metastasis of cancer by packaging and transferring bioactive constituents to recipient cells (Kalluri & McAndrews, 2023). Notably, almost all existing studies have mainly focused on EVs secreted from living cells, whereas the biological activities of dying cell‐released EVs in modulating cancer metastasis and chemoresistance have rarely been investigated. In this study, we found that EV‐Apo isolated from paclitaxel‐induced apoptotic breast cancer cells were enriched with CXCL1, which promoted the metastasis and chemoresistance of breast cancer by activating the TAM/PD‐L1 pathway. This finding was consistent with the existing reports that chemotherapy‐elicited EVs could promote the metastasis and chemoresistance of breast cancer. For example, Yang et al. reported that doxorubicin chemotherapy‐elicited EVs from breast cancer cells were enriched in miR‐378a‐3p and miR‐378d, which endowed chemoresistance by inducing WNT/β‐catenin and Notch stemness pathways. More meaningfully, the expression of the above miRNAs was also increased in the serum EVs of breast cancer patients receiving neoadjuvant chemotherapy, and was clinically associated with chemoresistance (Yang et al., 2021). Furthermore, Ioanna et al. reported that taxanes and anthracyclines chemotherapy‐elicited EVs from breast cancer cells were enriched in annexin‐A6, which has an important role in facilitating the establishment of lung metastasis by remodelling the pulmonary PMN (Keklikoglou et al., 2019). Carson et al. reported that doxorubicin chemotherapy upregulated both the PTX3 protein cargo in EVs and the secretion of EVs from breast cancer cells, finally resulting in the lung metastasis of breast cancer by priming the PMN (Wills et al., 2021). Both our study and previous reports have suggested that chemotherapy‐induced EV signalling is a promising target for developing novel cancer therapeutic approaches. Notably, our study specifically focused on EV‐Apo isolated from chemotherapy‐induced apoptotic breast cancer cells, whereas previous reports did not specify whether EVs originated from dead, dying, or surviving cells. Our findings are thus more instructive for the significance of dying cell‐released signalling. Furthermore, apart from focusing on EV cargo and secretion, we further revealed that paclitaxel treatment induced the biogenesis of ILVs in MVBs, which may account for the elevated secretion of EVs induced by chemotherapy. Moreover, we clearly showed that chemotherapy‐elicited EV‐Apo was predominantly phagocytosed by macrophages and therefore induced the establishment of pro‐metastatic TME, whereas previous reports mainly focused on the effect of chemotherapy‐elicited EVs on cancer cells. However, it should be noted that the present study only used paclitaxel as a chemotherapeutic drug since paclitaxel‐based chemotherapy is the first‐line treatment regimen for TNBC. Given the diversity of therapeutic strategies for breast cancer, the significance of EV‐Apo^CXCL1^ should be tested in other chemodrugs, as well as for other breast cancer subtypes.

CXCL1 is one of the most abundant chemokines in the TME and has multiple biological functions, which include carcinogenesis acceleration, neoangiogenesis promotion, metastasis, and chemoresistance induction (Korbecki et al., 2023; Wang et al., 2021). Clinicopathological studies have suggested that CXCL1 exhibits elevated expression in breast cancer tissue than in normal tissue and is closely correlated with advanced tumour stage, increased recurrence and decreased overall survival in patients with breast cancer (Korbecki et al., 2023; Zou et al., 2014). CXCL1 primarily exerts its chemotaxis function by specifically binding with the CXC chemokine receptor 2 (CXCR2) on recipient cells. For example, CXCL1 could accelerate the immune escape of breast cancer (Li et al., 2021) and non‐small cell lung cancer (Lv et al., 2014) by recruiting CXCR2^+^ Tregs to establish immunosuppressive TME. Additionally, CXCL1 could facilitate PMN formation in breast cancer (Zheng et al., 2020) and colorectal cancer (Wang et al., 2017) by recruiting CXCR2^+^ MDSCs. Apart from inducing the chemotaxis of immune cells, CXCL1 also directly promotes cancer metastasis and chemoresistance by inducing autophagy of cancer cells, activating self‐renewal of CSCs, and inducing the neoangiogenesis effects of endothelial cells (Korbecki et al., 2023; Wang et al., 2021). In this study, we reported that paclitaxel treatment significantly increased the CXCL1 protein cargo in EV‐Apo, which promoted the invasion and chemoresistance of breast cancer cells by polarizing M2 macrophages via transcriptionally upregulating their PD‐L1 expression. This result not only enriches the existing understanding of the pro‐tumour mechanism of CXCL1 from the novel perspective of EVs but also provides experimental evidence for the approved combination regimen of nab‐paclitaxel and a PD‐L1 monoclonal antibody (e.g., atezolizumab) in patients with advanced TNBC (Schmid et al., 2018; Schmid et al., 2020). Consistent with our findings, other chemodrugs, including doxorubicin, carboplatin, oxaliplatin, and gemcitabine, have been reported to induce the secretion of CXCL1 from cancer cells and therefore to mediate the emergence of cancer chemoresistance (Korbecki et al., 2023). Additionally, Seifert *et al.* reported that gemcitabine‐elicited necroptosis CXCL1 signals also accelerated pancreatic oncogenesis and progression by inducing immunosuppressive TAMs via activating PD‐L1 expression (Seifert et al., 2016). The aforementioned results suggest that CXCL1 is an important molecule linking chemotherapy‐induced cell death and cancer chemoresistance. Additionally, our results also revealed novel biological activities of the chemotherapy‐elicited CXCL1 signal in EV‐Apo biogenesis. CXCL1 blockage significantly hindered the paclitaxel‐induced biogenesis and release of EV‐Apo, whereas the addition of exogenous CXCL1 partially counteracted the inhibitory impact of BHS on this process. To the best of our knowledge, there have been rare reports focusing on this. However, clinical evidence is urgently needed to demonstrate the significance of the EV‐Apo^CXCL1^ signal in predicting breast cancer chemoresponse.

The development of small molecules enhancing the pCR rate is critical for improving the clinical outcome of TNBC. In recent years, the emergence of PD‐L1 immune checkpoint inhibitors (such as pembrolizumab and atezolizumab) significantly increased the pCR rate and prolonged progression‐free survival in TNBC patients (Park et al., 2020). However, immune checkpoint inhibitors are expensive and usually induce adverse effects, which severely limits their clinical applications. Herein, it was found that BHS, a natural compound, significantly synergized with paclitaxel to inhibit breast tumour growth and lung metastasis. This finding is consistent with the previous reports that BHS synergizes chemotherapy in multiple malignancies. For example, BHS inhibited cisplatin resistance in ovarian cancer by suppressing autophagy via downregulating the HIF‐1α/ATG5 axis (Zhou et al., 2023). BHS also potentiated paclitaxel chemotherapy in human melanoma by inducing apoptosis via inhibiting the TLR4 pathway (Wu et al., 2012). Differently from the above studies, our investigation revealed the chemosensitising effect of BHS from the new perspective of EV‐Apo. BHS significantly inhibited TAM infiltration and PD‐L1 expression induced by chemotherapy‐elicited EV‐Apo. Mechanistically, BHS not only decreased the CXCL1 protein cargo in EV‐Apo but also inhibited the biogenesis of ILVs in MVBs. Specifically, BHS bound the LEU104 residue of the FLOT2 protein and led to a binding interruption between RAB31 and FLOT2. This was consistent with previous reports that the ^104^LQTL^107^ motif in the SPFH domain of FLOT2 is responsible for its interaction with RAB31 to drive ILV biogenesis (Wei et al., 2021). The above finding uncovered novel biological activities of BHS in regulating the EV‐Apo cargo and biogenesis, which is of interest for drug development targeting EV modulation. Additionally, in our previous study, we found that BHS exhibited minimal observable embryotoxicity or teratogenic effects on zebrafish embryos even at a high in vitro concentration of 20 µM. Furthermore, BHS administration at a dose of 20 mg/kg/d exhibited little hepatotoxicity or nephrotoxicity in mice (Wang et al., 2020). These encouraging pre‐clinical results highlighted the low toxicity of BHS. As a natural prenylflavonoid, BHS is abundant in the *Epimedium* herb and can be biosynthesized via simple synthetic routes. Notably, BHS is a metabolite of icariin, characterized by loss of the glycosyl moiety at the C‐7 position, and is thus also known as icariside II. In 2022, icaritin capsules were approved by the National Medical Products Administration of China for the clinical treatment of hepatocellular carcinoma. Therefore, BHS may be promising to be developed as a chemosensitising adjuvant due to its advantages of effectiveness, low toxicity, and easy availability. Despite these encouraging advantages, more preclinical studies and clinical trials are still warranted to fully understand the chemosensitising activities of BHS before its clinical applications.

## 5 CONCLUSION

Taken together, the results of this study shed new light on EV‐Apo^CXCL1^ as a novel therapeutic target to chemosensitise TNBC and highlight BHS as a promising chemotherapy adjuvant by disrupting EV‐Apo^CXCL1^ biogenesis via directly disturbing the RAB31/FLOT2 interaction.

## AUTHOR CONTRIBUTIONS

**Shengqi Wang**: Conceptualization; data curation; formal analysis; funding acquisition; investigation; methodology; resources; software; validation; visualization; writing—original draft; writing—review and editing. **Jing Li**: Data curation; formal analysis; investigation; methodology; validation; visualization. **Shang Xu**: Data curation; formal analysis; investigation; methodology; validation. **Neng Wang**: Resources. **Bo Pan**: Methodology; software; visualization. **Bowen Yang**: Methodology. **Yifeng Zheng**: Methodology. **Juping Zhang**: Methodology. **Fu Peng**: Funding acquisition. **Cheng Peng**: Funding acquisition; supervision. **Zhiyu Wang**: Conceptualization; project administration; resources; supervision; validation; visualization; writing—original draft; writing—review and editing.

## CONFLICT OF INTEREST STATEMENT

The authors declare no conflict of interest.

## Supporting information

Supporting Information

## Data Availability

All data generated or analysed during this study are included either in this article or in the supplementary information files.
