# Supplementary material for: Baohuoside I chemosensitises breast cancer to paclitaxel by suppressing extracellular vesicle/CXCL1 signal released from apoptotic cells
Source: J Extracell Vesicles. 2024 Jul 25;13(7):e12493. doi: 10.1002/jev2.12493 (PMC11270583; doi:10.1002/jev2.12493)
Supplement: Supplementary file 1 — Supporting Information [file JEV2-13-e12493-s001.docx]

**Supplementary Figures**

**
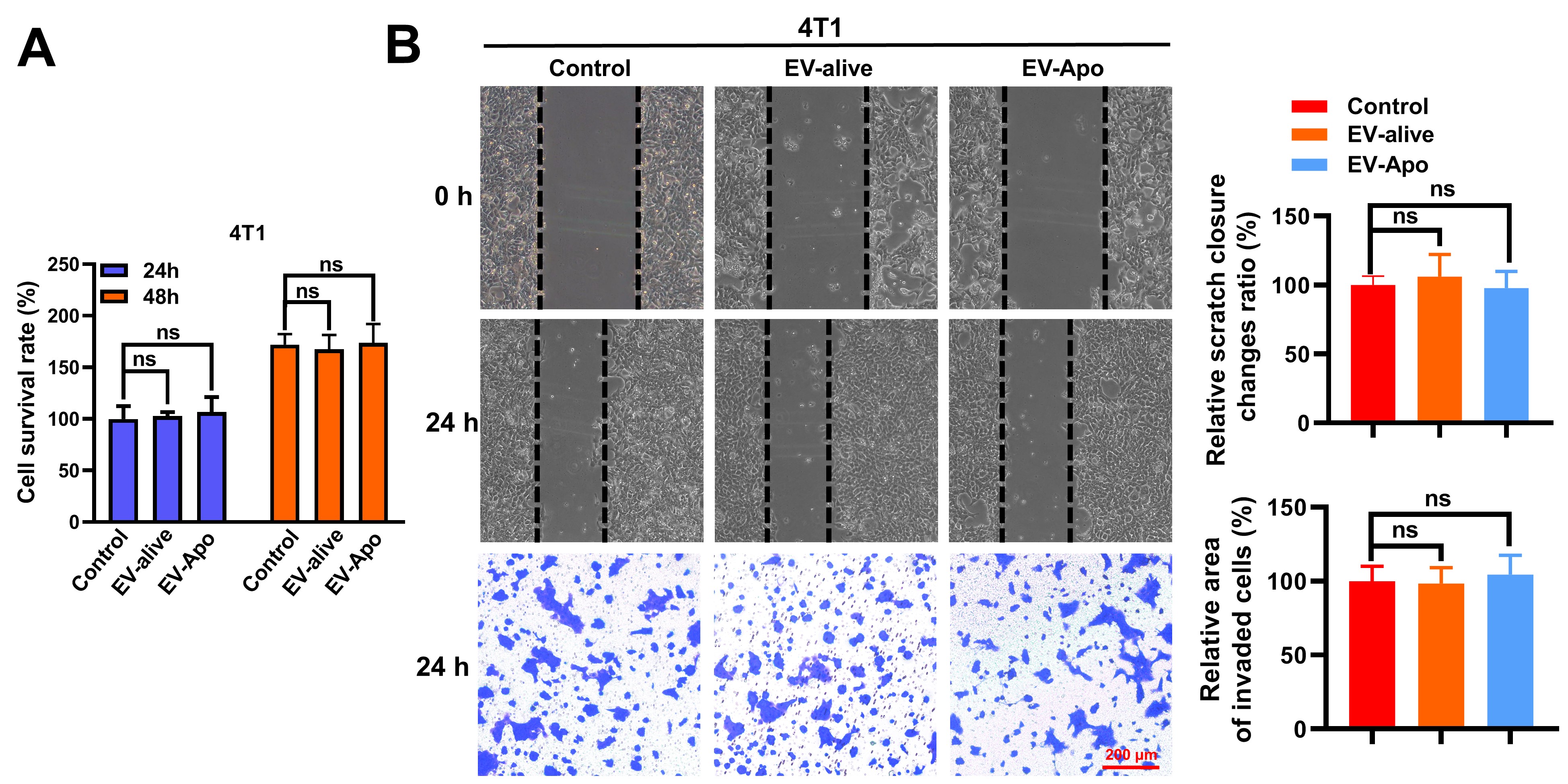
**

**Supplementary Figure 1. Effects of EV-Apo and EV-alive on the malignant biological behaviors of breast cancer cells in monoculture.** Both EV-Apo and EV-alive (100 μg/ml) exhibited little effect on the proliferation, migration, and invasion of 4T1 cells in monoculture. n = 3.


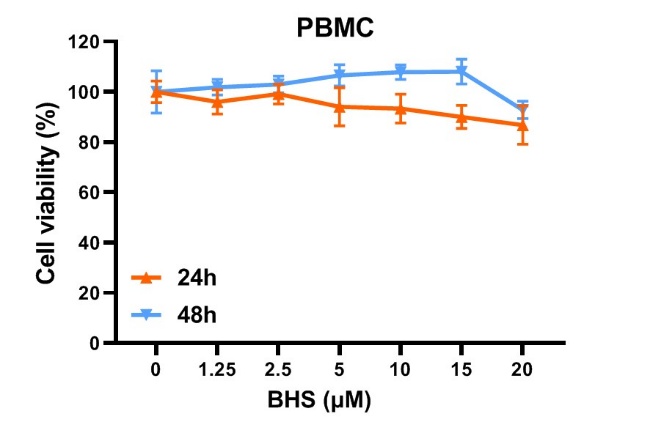


**Supplementary Figure 2. The cytotoxic effect of BHS on mouse PBMCs.** Cytotoxicity of BHS in mouse PBMCs was detected using the CCK8 assay. n = 6.


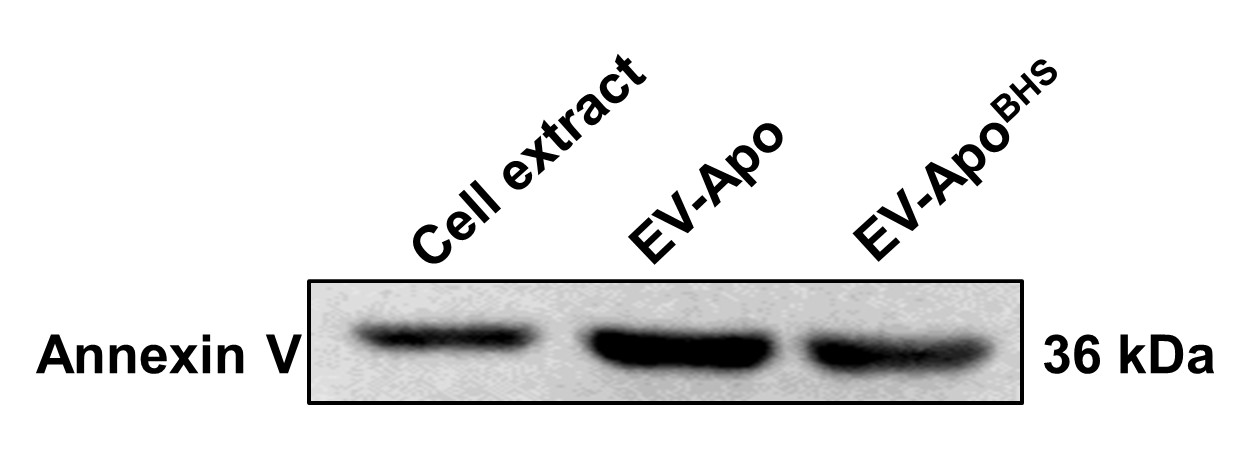


**Supplementary Figure 3. Comparison of Annexin V expression levels among 4T1 cell extract, EV-Apo, and EV-Apo^BHS^.** Equal amounts of cell samples and EVs (30 μg) were subjected to immunoblot analysis detecting the apoptotic marker Annexin V. n = 3.

**
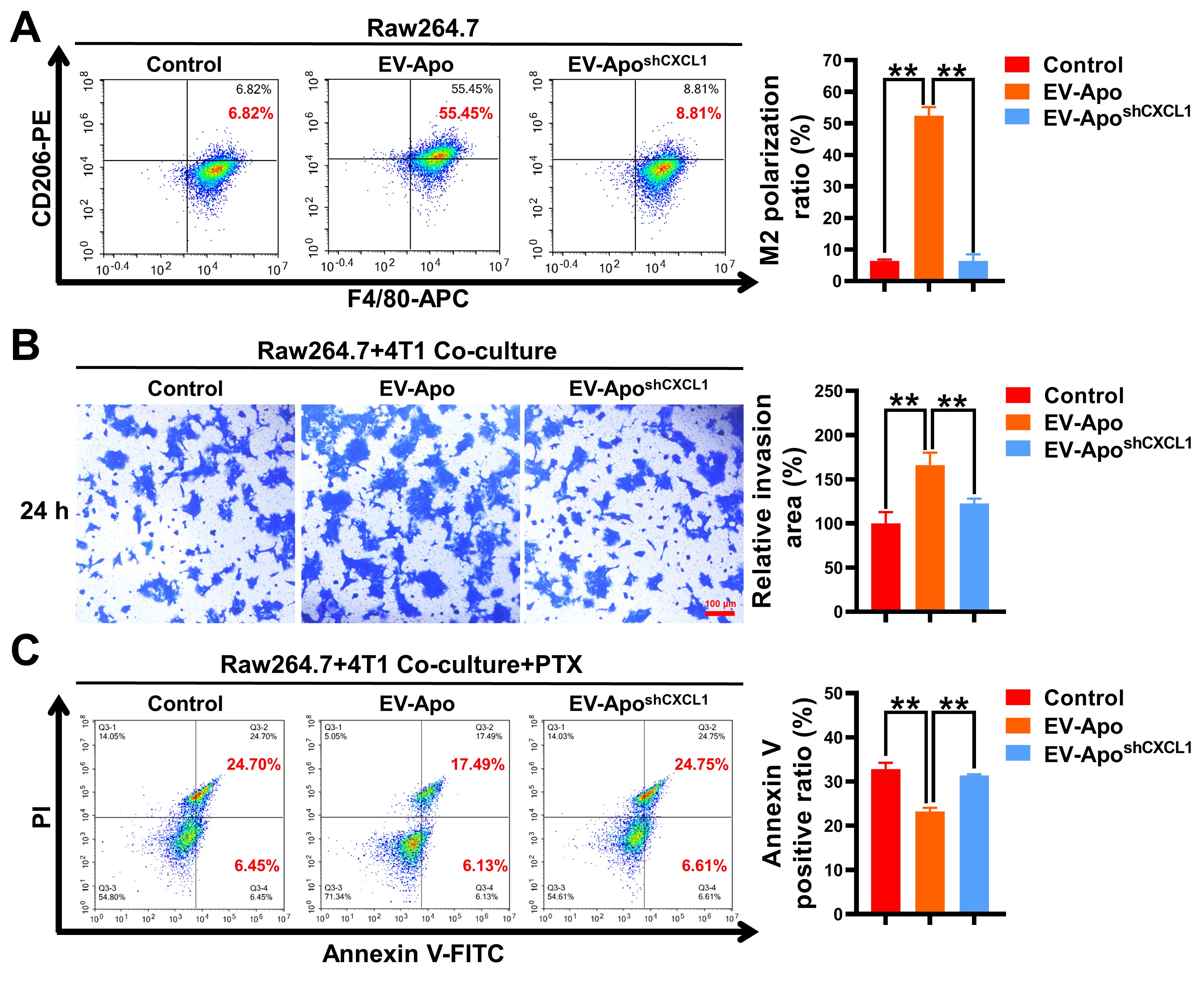
**

**Supplementary Figure 4. Effect of EV-Apo/CXCL1 on macrophage M2 polarization as well as breast cancer invasion and apoptosis resistance.** **(A)** M2 polarization changes of Raw264.7 macrophages after treatments as indicated for 48 h were detected by an F4/80^+^/CD206^+^ population analysis. **(B–C)** Changes in invasion (B) and apoptosis resistance (C) of the co-cultured 4T1 cells after treatments as indicated for 24 h or 48 h. EVs were used at a concentration of 100 μg/ml. n = 3. ^**^*p* < 0.01.


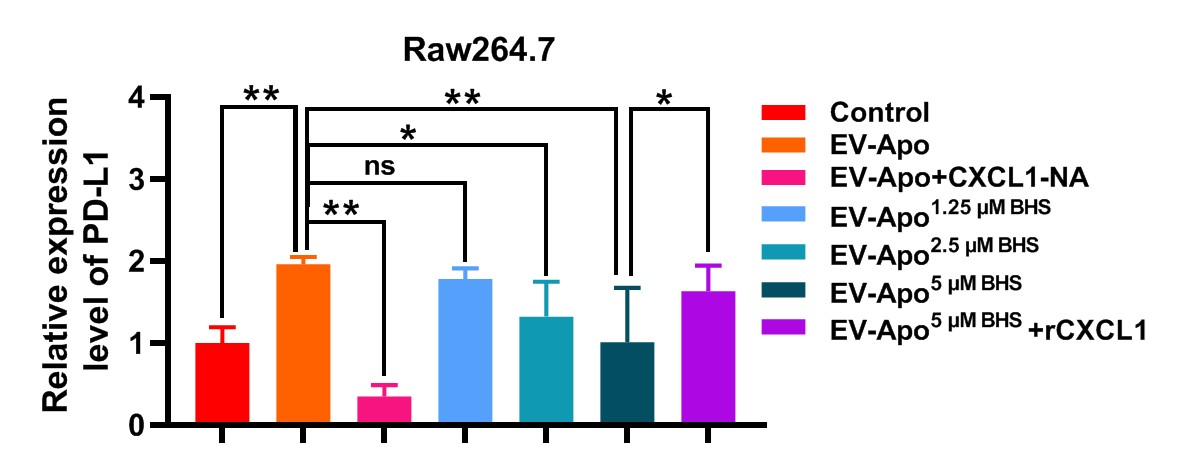


**Supplementary Figure 5. A histogram comparing the differences in PD-L1 expression levels in Figure 4E.** n = 3. ^*^*p* < 0.05, ^**^*p* < 0.01.


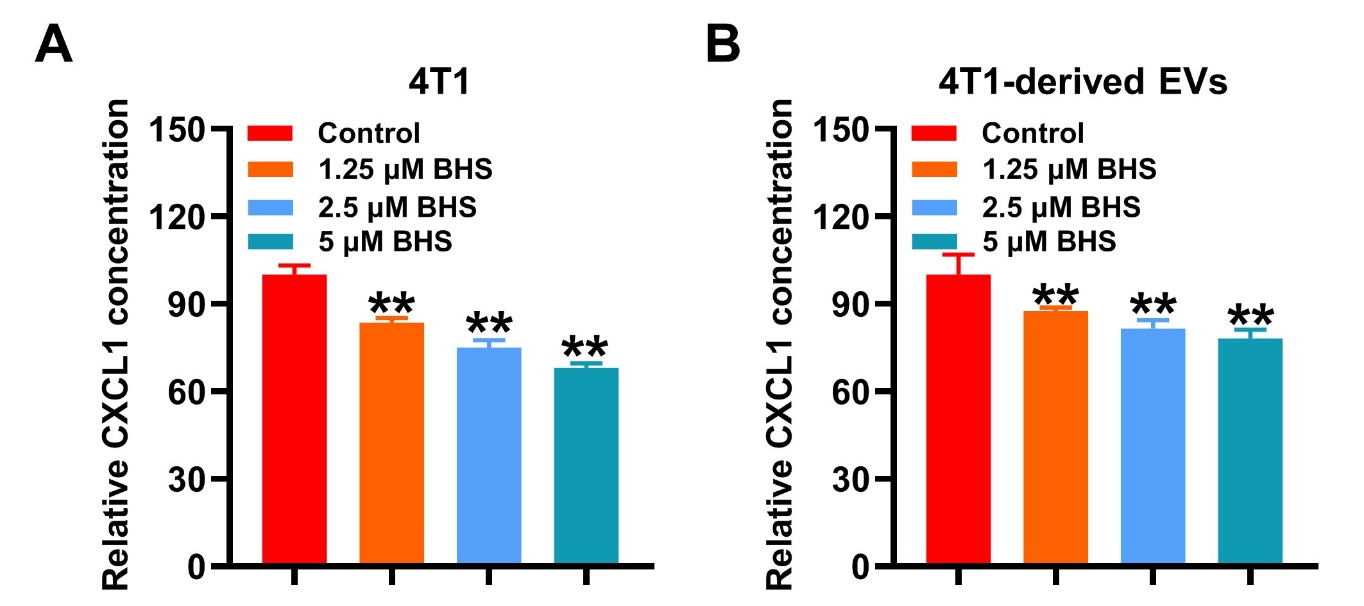


**Supplementary Figure 6. Effect of BHS on CXCL1 secretion from 4T1 cells and CXCL1 cargo level in 4T1 cell-derived EVs.** 4T1 cells were treated with BHS as indicated for 48 h, and CXCL1 level was detected by ELISA assay. n = 3. ^**^*p* < 0.01.


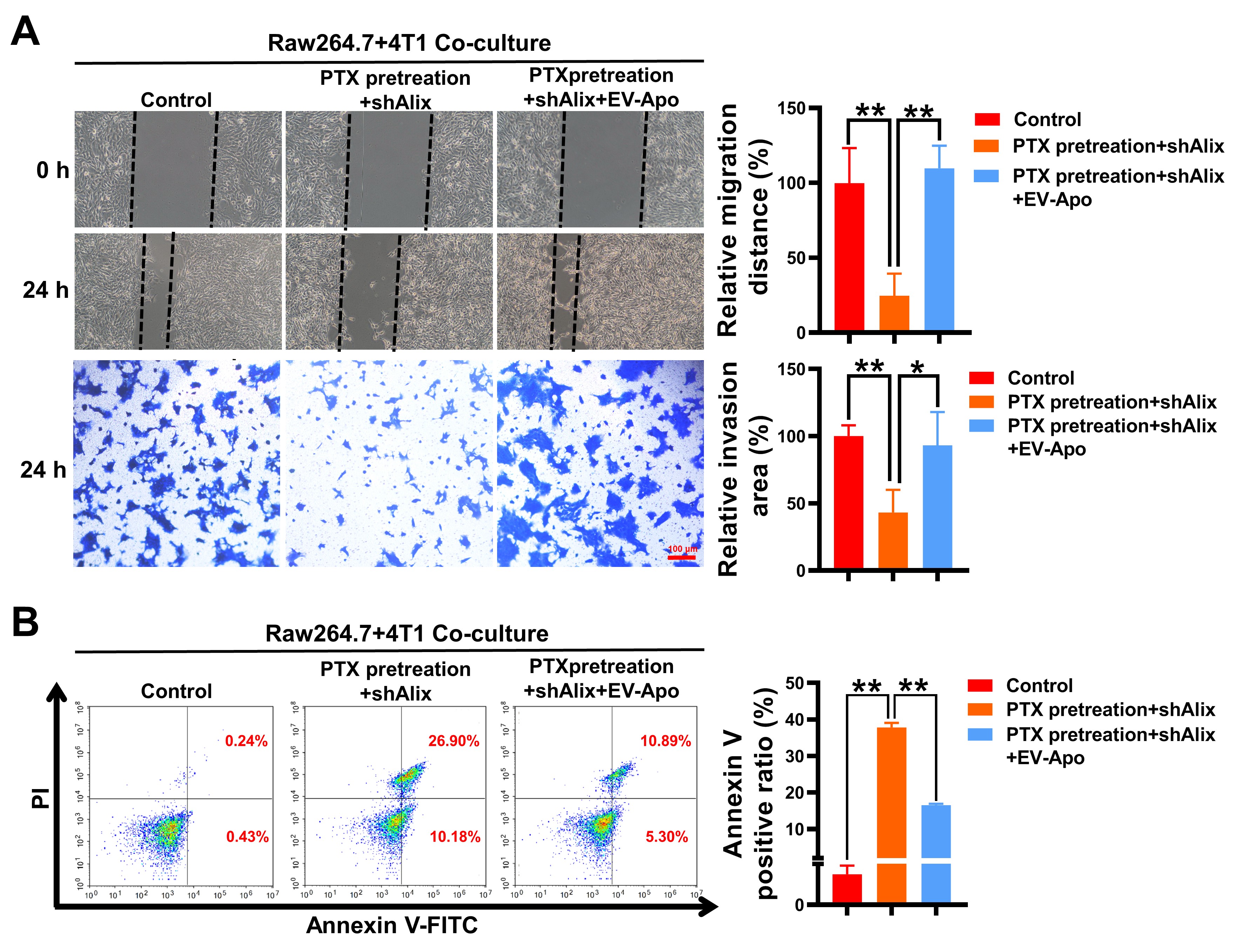


**Supplementary Figure 7. EV-Apo addition rescues the effects of Alix knockdown on paclitaxel-induced cell death and the inhibition of migration/invasion.** Changes in migration, invasion and apoptosis resistance of the co-cultured 4T1 cells after treatments as indicated for 24 h or 48 h. EVs were used at a concentration of 100 μg/ml. n = 3. ^*^*p* < 0.05, ^**^*p* < 0.01.

**
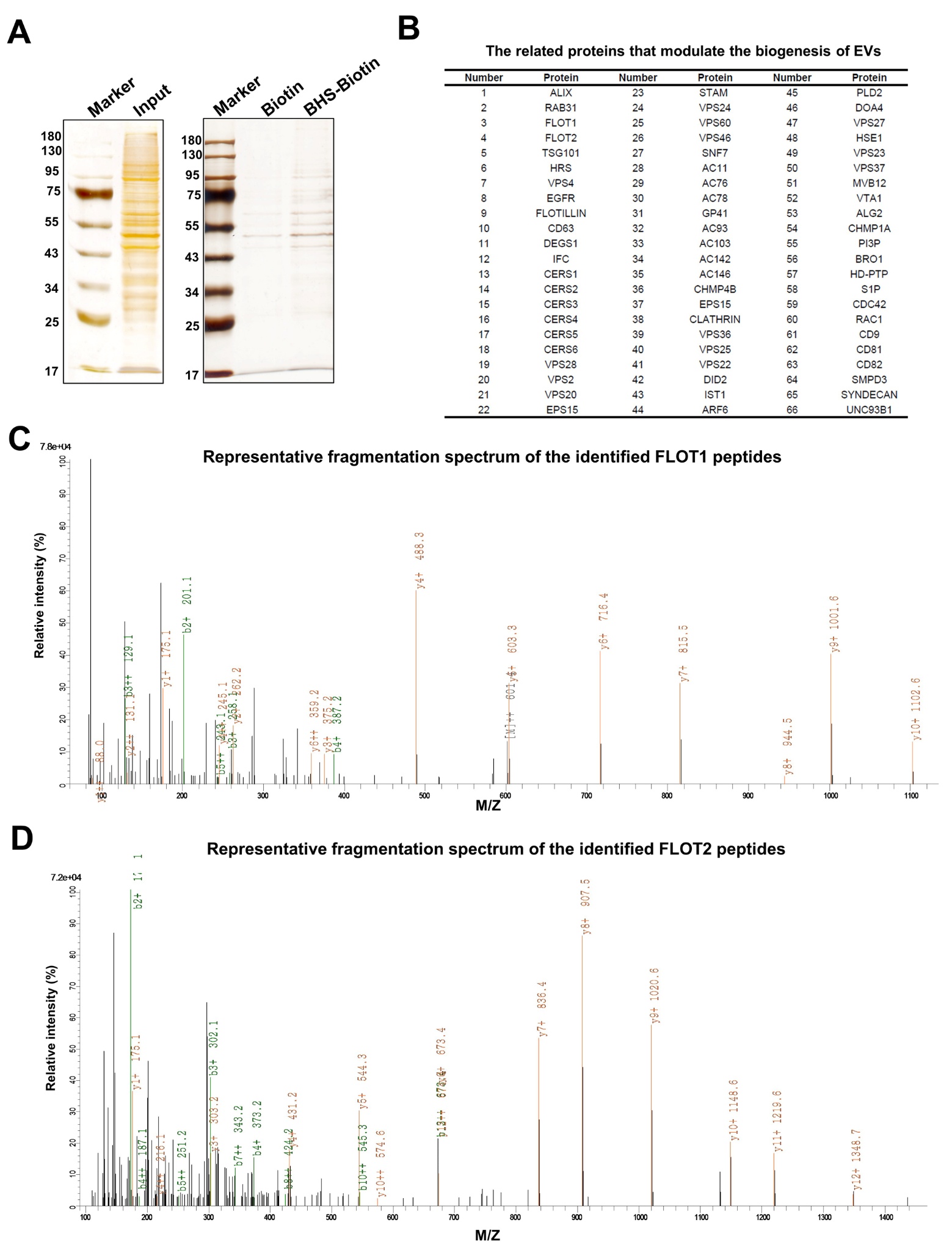
**

**Supplementary Figure 8. Identification of the target protein of BHS in modulating EV biogenesis. (A)** A silver staining analysis of the input samples and pull-down samples. **(B)** Related proteins that modulate the biogenesis of EVs. **(C-D)** A representative fragmentation spectrum of the identified FLOT peptides.


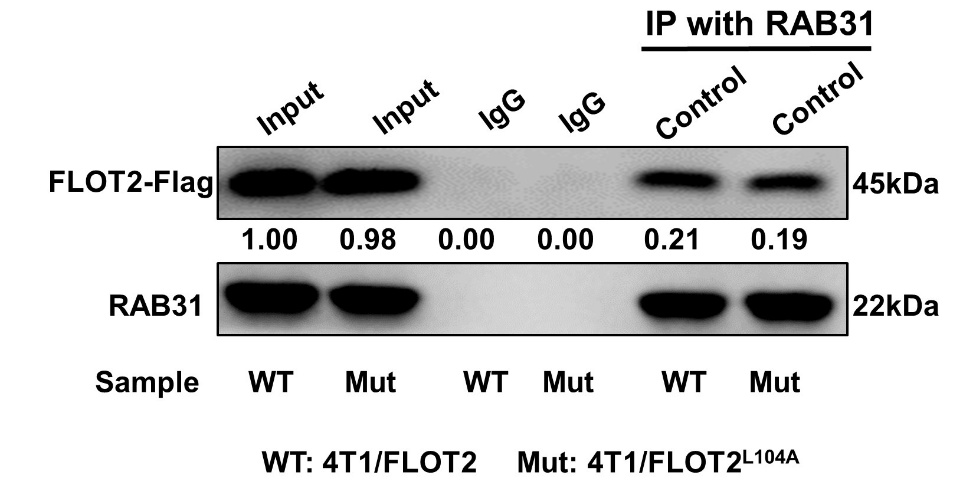


**Supplementary Figure 9. FLOT2^L104A^ mutant retains binding to RAB31**. A Co-IP analysis of the interaction activity difference between RAB31 and FLOT2-Flag in 4T1/FLOT2 cells and 4T1/FLOT2 ^L104A^ mutant cells. n = 3.


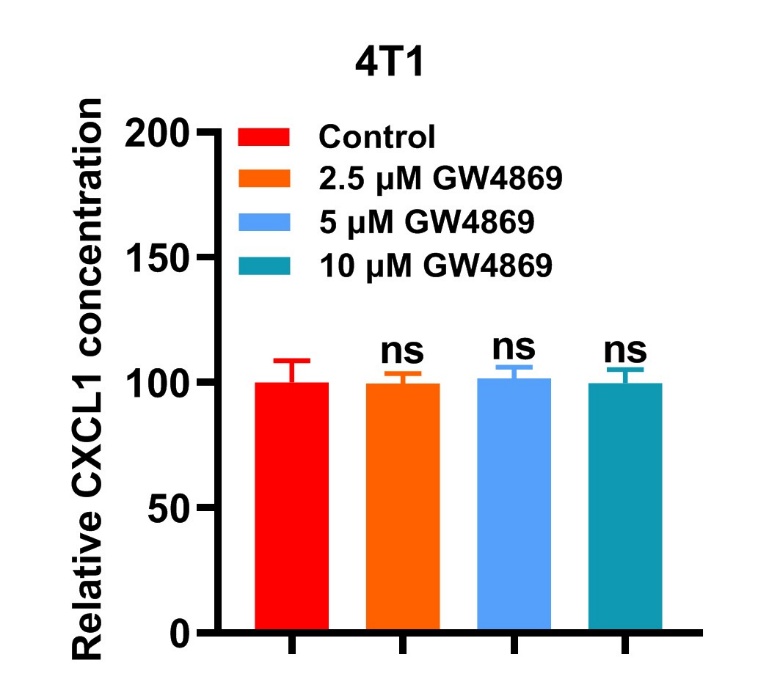


**Supplementary Figure 10. Effect of GW4869 on CXCL1 secretion from 4T1 cells.** 4T1 cells were treated with GW4869 as indicated for 48 h, and CXCL1 level was detected by ELISA assay. n = 3.

**
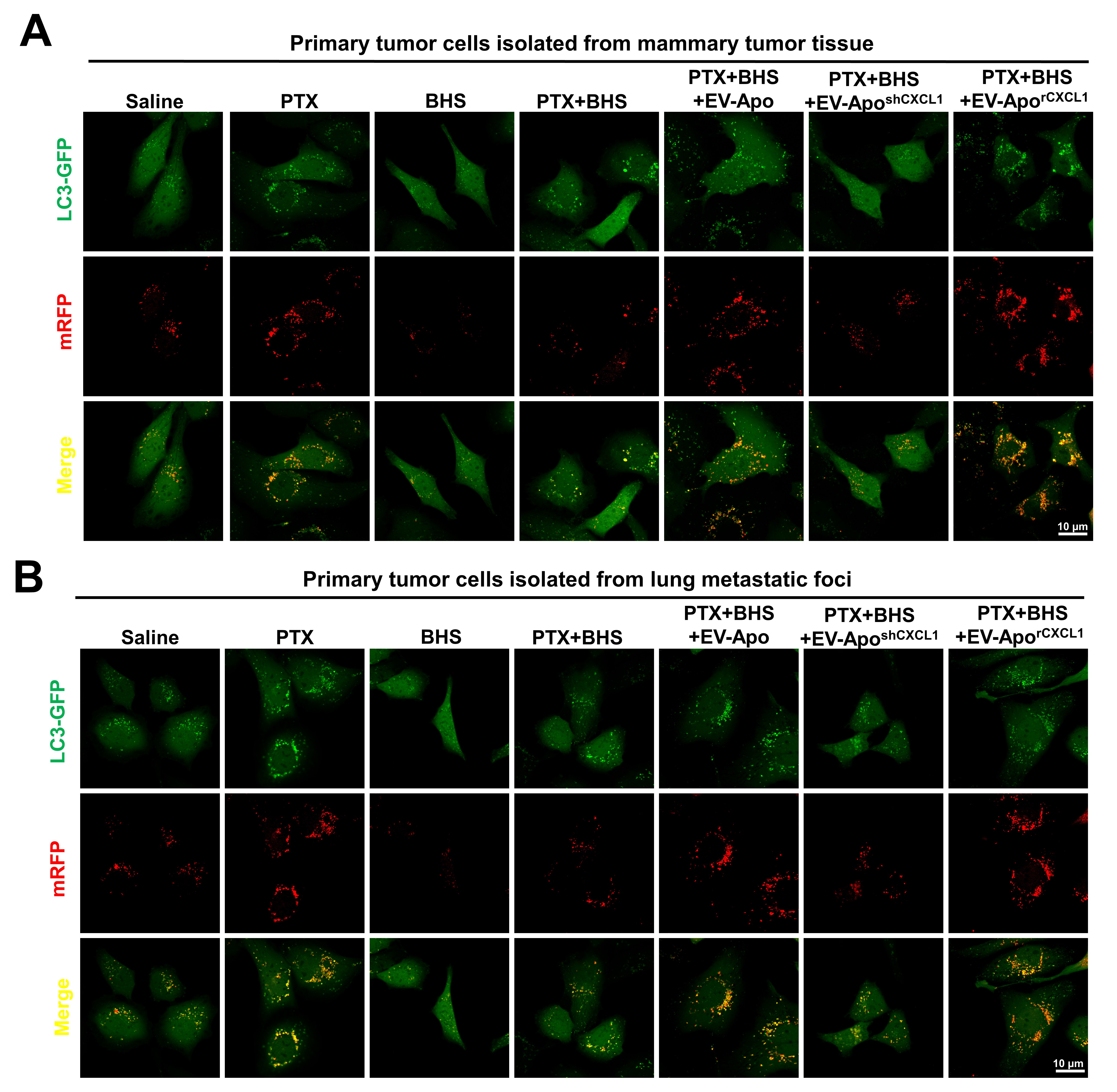
Supplementary Figure 11. The autophagic activity of the primary tumor cells isolated from both tumor tissues and lung metastatic foci.** Autolysosomes were stained with mRFP (red). Autophagosomes were characterized by yellow fluorescence (colocalization of mRFP and GFP). n = 3.
